# Prognostic Biomarkers for Gastric Cancer: An Umbrella Review of the Evidence

**DOI:** 10.3389/fonc.2019.01321

**Published:** 2019-11-29

**Authors:** Cen Zhou, Xi Zhong, Yongxi Song, Jinxin Shi, Zhonghua Wu, Zhexu Guo, Jie Sun, Zhenning Wang

**Affiliations:** Department of Surgical Oncology and General Surgery, Key Laboratory of Precision Diagnosis and Treatment of Gastrointestinal Tumors, Ministry of Education, The First Affiliated Hospital of China Medical University, Shenyang, China

**Keywords:** biomarkers, gastric cancer, umbrella review, prognostic, survival

## Abstract

**Introduction:** Biomarkers are biological molecules entirely or partially participating in cancerous processes that function as measurable indicators of abnormal changes in the human body microenvironment. Aiming to provide an overview of associations between prognostic biomarkers and gastric cancer (GC), we performed this umbrella review analyzing currently available meta-analyses and grading the evidence depending on the credibility of their associations.

**Methods:** A systematic literature search was conducted by two independent investigators of the PubMed, Embase, Web of Science, and Cochrane Databases to identify meta-analyses investigating associations between prognostic biomarkers and GC. The strength of evidence for prognostic biomarkers for GC were categorized into four grades: strong, highly suggestive, suggestive, and weak.

**Results:** Among 120 associations between prognostic biomarkers and GC survival outcomes, only one association, namely the association between platelet count and GC OS, was supported by strong evidence. Associations between FITC, CEA, NLR, foxp3+ Treg lymphocytes (both 1- and 3-year OS), CA 19-9, or VEGF and GC OS were supported by highly suggestive evidence. Four associations were considered suggestive and the remaining 108 associations were supported by weak or not suggestive evidence.

**Discussion:** The association between platelet count and GC OS was supported by strong evidence. Associations between FITC, CEA, NLR, foxp3+ Treg lymphocytes (both 1- and 3-year OS), CA 19-9, or VEGF and GC OS were supported by highly suggestive evidence, however, the results should be interpreted cautiously due to inadequate methodological quality as deemed by AMSTAR 2.0.

## Introduction

Gastric cancer (GC) was the most common cancer worldwide less than a century ago (1). Despite a decreasing incidence in recent decades, GC remains the most commonly diagnosed cancer in Eastern Asia (2). According to the National Central Cancer Registry in China, GC is the second most common cancer in china, with 298,800 cases in 2013 alone, which means that approximately 42 individuals suffer GC in every 100,000 people (3). The best options to reduce mortality are treatments aimed at early detection, systematic prevention and personalized therapy. Meanwhile, traditional treatment strategies such as surgery have potentially reached a ceiling regarding locoregional control and mortality reduction, reflecting the dilemma that GC remains unsatisfactorily incurable worldwide (4).

Biomarkers are biological molecules entirely or partially participating in cancerous processes that function as measurable indicators of abnormal changes in the human body microenvironment (5, 6). Many studies have reported the importance of biomarkers in clinical GC applications including diagnosis, treatments and prognosis. There are currently three main types of cancer biomarkers distinguished by clinical use: predictive, prognostic, and pharmacodynamic markers (7–10). Countries with high GC incidence, such as Japan, have established adequate tumor monitoring systems to detect and diagnose GC at early stages, greatly improving survival (4). Prognostic biomarkers play essential roles in distinguishing between benign and malignant tumors, monitoring progress of advanced GCs, and predicting survival outcomes. Several protein cancer biomarkers are widely used and have become routine in clinical practice, especially α-fetoprotein (AFP) which has been proven to improve early diagnosis of hepatocellular cancer, resulting in more superior survival outcomes (11, 12). Many cohort and case-control studies have explored biomarkers associated with GC, and several meta-analyses have been published to systematically analyze these results. Aiming to provide an overview of associations between prognostic biomarkers and GC, we performed this umbrella review analyzing currently available meta-analyses and grading the evidence depending on the credibility of their associations.

## Methods

### Search Strategy and Eligibility Criteria

A systematic literature search was conducted by two independent investigators of the PubMed, Embase, Web of Science, and Cochrane Databases to identify meta-analyses investigating associations between prognostic biomarkers and GC published from inception through April 11, 2019. The following relevant keywords were used to conduct our electronic database search: (risk factors OR Helicobacter pylori OR H. pylori OR peptic ulcer disease OR gastritis OR inflammation OR IL-7 OR IL-10 OR gastric ulcer OR gastroesophageal reflux disease OR GERD OR esophagogastric junction OR dysplastic intestinal metaplasia OR cardia OR smoking OR smoker OR alcohol OR chemical exposure OR occupational exposure OR high temperature OR particulates OR metal OR chromium OR asbestos OR talc OR crystalline silica OR diet OR salt OR preserved meat OR red meat OR coffee OR caffeinated intake OR caffeine intake OR caffeine OR decaffeinated OR decaffeinated intake OR fruits OR vegetables OR obesity OR obese OR BMI OR body mass index OR anemia OR gastric surgery OR radiation OR Epstein-Barr virus OR EBV OR socioeconomic status OR poverty OR wealth OR education OR level of education OR educational level OR schooling OR blood group OR blood type OR sex OR gender OR sexuality OR man OR male OR woman OR female OR anti-estrogen drugs OR tamoxifen OR hormone replacement therapy OR HRT OR parity OR pregnancy OR menopause OR premenopausal OR post-menopausal OR ethnic origin OR ethnicity OR race OR screening programs OR radiography OR endoscopy OR serum pepsinogen level OR exercise OR physical activity OR family history OR familial OR radiation OR radiotherapy OR cohabiting OR living together OR partner OR partnered OR insulin OR metformin OR aspirin OR aspirin containing medications OR drugs OR medicine) AND (gastric cancer OR gastric carcinoma OR gastric neoplasia OR gastric tumor OR gastric neoplasm OR gastric maligna^*^ OR GC OR stomach carcinoma OR stomach neoplasia OR stomach tumor OR stomach neoplasm OR stomach maligna^*^) AND (systematic review OR meta-analysis OR metaanalysis). Only meta-analyses were included in this umbrella review, irrespective of publication year or language; case reports, commentaries, editorials, conference abstracts and letters were excluded. We also manually reviewed the reference lists in the retrieved meta-analyses to include any related studies.

A detailed eligibility criterion was formulated for study inclusion: (1) we included studies clearly examining associations between prognostic biomarkers, rather than predictive or pharmacodynamic markers, and GC survival outcomes including but not limited to overall survival (OS), disease-free survival (DFS), progression-free survival (PFS) and cancer-specific survival (CSS). (2) We excluded studies investigating genetic polymorphism and GC incidence. Studies focusing on benign gastric tumors such as leiomyoma, neurofibroma and gastrointestinal stromal tumors were also excluded (3). We excluded meta-analyses containing less than three original studies or not providing sufficient data from each individual study. When two or more meta-analyses focused on one specific association, we included the meta-analysis with largest sample size.

### Data Extraction

Two investigators independently performed data extraction from included meta-analyses and resolved differences through discussion. The following values were retrieved from each included study: first author name, publication year; country, name and classification of biomarker and its associations with GC, relative risk estimates, including risk ratio (RR), odds ratio (OR), hazard ratio (HR) and the corresponding 95% confidence interval (CI), number of include studies, number of cases, and population size.

### Quality Assessment

The methodological quality of included meta-analyses was evaluated through AMSTAR (A MeaSurement Tool to Assess systematic Reviews) version 2.0 (2017) (13), a vital appraisal tool for umbrella reviews to assess involved randomized trials with high efficiency. The revised version simplifies response categories and contains 16 items in all which provide a more comprehensive appraisal compared with the original AMSTAR. Rather than outputting an overall score, AMSTAR 2 evaluates single study quality by calculating scores in specific items and then describes results as either high, moderate, low, or critically low grade.

### Statistical Analysis

Statistical analyses were conducted using STATA version 12.0 (StataCorp. LLC, College Station, TX, USA). Random-effect models were used to estimate summary effects for included studies considering the inevitable heterogeneity caused by multiple sources. Relative risk estimates, 95% confidence interval (CI) and corresponding *P*-values were calculated. The significance level was set to *P* < 0.05 (14).

Interstudy heterogeneity was analyzed through Cochran's Q test and the *I*^2^ statistic was calculated. Ranging from 0 to 100%, *I*^2^ quantitatively demonstrates variability among risk estimates, with *I*^2^ > 50% indicating great heterogeneity (15). Interstudy heterogeneity was also analyzed using 95% prediction intervals (PI), assessing the impact of uncertainty in individual studies and prone to be more conservative (16, 17).

Several methods were used to evaluate bias in associations between prognostic biomarkers and GC. Egger's regression asymmetry test was performed to assess whether small-study effects existed (17), with a *P* < 0.01 considered statistically significant with more conservative results in the largest study.

Excess significance bias was applied to avoid potential biases such as selective reporting biases or publication biases. To assess whether the number of expected studies (E) was in accordance with the observed number (O) with nominally significant results or less, chi-square statistics were performed (18) with a two-tailed *P* < 0.10 as the statistical significance threshold. The number of studies expected to be statistically significant was calculated by summing up statistical power estimates extracted from each component using an algorithm from a non-central t distribution. The observed number was extracted from the relative risk estimate of the largest study. In cases where O > E and *P* < 0.10, excess significance was considered positive.

Credibility ceiling sensitivity analyses were performed for weak evidence to skeptically analyze precise results provided by included meta-analyses. The credibility ceiling was set at 10% for this study, based on the assumption that the likelihood of a specific effect always has a limitation, in other words, no matter how well-designed a study was, its effect in this particular aspect is restricted and impossible to exceed maximum value (19).

### Strength of Existing Evidence

The strength of evidence for prognostic biomarkers for GC were categorized into four grades in accordance with previous studies (20, 21): strong, highly suggestive, suggestive, and weak. Categorization criteria are as follow: (1) a study was considered as strong evidence if it presented a *P* < 10^−6^, *I*^2^ < 50%, calculated 95% PI excluding the null value, a sample size >1,000 cases, was absent evidence of small-study effects and excess significance and survived the 10% credibility ceiling (*P* > 0.05); (2) a study would be rated as highly suggestive evidence if it presented a *P* < 10^−6^ with a sample size >1,000 cases; (3) a study would be categorized as suggestive evidence if it presented a *P* < 10^−3^ with a sample size >1,000 cases; (4) a study would be assessed as weak evidence if it presented a *P* < 0.05.

## Results

### Characteristics of the Included Systematic Reviews and Meta-Analyses

**Figure 1:**
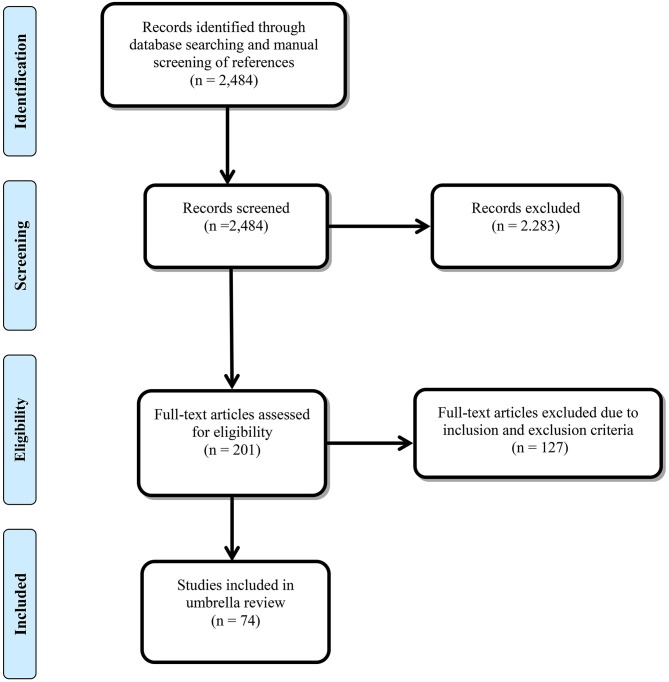
Flow diagram.

A total of 2,484 records were identified from the literature search and manual screening of references, of which 2,283 were excluded after title and abstract screening. Ultimately, 74 of the remaining 201 studies met the inclusion criteria after full-text review (22–97). The search flowchart is shown in Figure 1, and the full list of the 201 studies and exclusion reasons for 127 of them are shown in Supplementary Table S1. Of note, we selected the most recent systematic review and meta-analysis investigating the association between HER2 and GC mortality (96) rather than the study with the largest number of primary studies (98) for inclusion because the latter searched for studies published in 2015 while the former was published in 2017 and the included studies needed to be updated. The included studies covered 120 different associations between prognostic biomarkers and GC survival outcomes, more than 79,000 subjects, and over 1,000 studies. Characteristics of the 120 associations in the included systematic reviews and meta-analyses are shown in Table 1. Data on the primary studies included in the 74 systematic reviews and meta-analyses were also extracted, processed, and coded to perform various analyses.

**Table 1:** Characteristics of the 120 associations in the included systematic reviews and meta-analyses.

**References**	**Biomarker**	**Association between biomarker and gastric cancer**	**Effect metrics**	**Country**	**No. of study estimates**	**No. of cases/total population**	**Summary relative risk estimate (95% CI)**
Kim et al. (25)	ARID1A	OS	HR	Korea	4	344/1,316	1.51 (1.25–1.82)
Liu et al. (88)	BIRC5	OS	HR	China	18	492^#^/1,528^#^	1.15 (0.82–1.61)
Chen et al. (82)	BIRC5	5-year OS	OR	China	6	230^#^/634	1.61 (1.41–1.85)
	PTEN	5-year OS	OR	China	9	639^#^/1,548	1.59 (1.38–1.84)
	HIF-1α	5-year OS	OR	China	10	454/1,400	1.52 (1.28–1.81)
Shao et al. (75)	Bmi-1	OS	HR	China	3	396^#^/633	1.50 (1.22–1.85)
Song et al. (57)	CA 19-9	OS	HR	China	29	2609^#^/8882	1.83 (1.56–2.15)
		DFS	HR	China	7	497^#^/2037	1.86 (1.17–2.96)
		DSS	HR	China	6	473^#^/1304	1.30 (1.04–1.61)
Du et al. (37)	CCR7	5-year OS	HR	China	4	94^#^/569	0.47 (0.31–0.70)
Lu et al. (41)	CD44	5-year OS	HR	China	9	653/1234	1.87 (1.55–2.26)
	CD133	5-year OS	HR	China	8	901/1424	2.15 (1.71–2.70)
Jiang et al. (26)	CD3+ T lymphocytes	OS	HR	China	11	826^#^/1851	0.66 (0.54–0.80)
	CD4+ T lymphocytes	OS	HR	China	9	655^#^/1762	0.80 (0.64–1.00)
	CD8+ T lymphocytes	OS	HR	China	13	1012/2185	0.83 (0.70–0.99)
	Foxp3+ Treg lymphocytes	OS	HR	China	20	1147/2725	0.97 (0.74–1.28)
	Dendritic cells	OS	HR	China	3	149/402	0.62 (0.15–2.51)
Wu et al. (54)	CD44	OS	HR	China	9	594/1210	0.91 (0.59–1.41)
		DFS	HR	China	3	121/286	1.68 (1.14–2.49)
	CD44v6	OS	HR	China	5	154^#^/441	1.26 (0.33–4.84)
Lu et al. (42)	CD44v6	5-year OS	OR	China	5	394/796	1.41 (0.80–2.49)
Meng et al. (60)	CDH17	5-year OS	RR	China	6	456^#^/1716	0.87 (0.67–1.14)
Wang et al. (92)	Cdx2	5-year OS	HR	China	4	199/475	2.21 (1.78–2.74)
Deng et al. (65)	CEA	OS	HR	China	51	3491^#^/8519	1.73 (1.57–1.90)
		DFS	HR	China	6	295^#^/1535	2.27 (1.72–3.01)
		DSS	HR	China	7	542/1227	1.95 (1.50–2.54)
Liu et al. (94)	Tissue VEGF	OS	HR	China	21	1056^#^/2691	2.13 (1.71–2.64)
		DFS	HR	China	7	465/1114	2.03 (1.57–2.62)
		DSS	HR	China	3	190/381	2.59 (1.33–5.06)
	Circulating VEGF	OS	HR	China	3	105/209	4.22 (2.47–7.18)
	Tissue VEGF-D	OS	HR	China	4	99^#^/282	1.73 (1.25–2.40)
Liu et al. (62)	CLDN4	OS	HR	China	7	378^#^/1030	2.01 (1.62–2.50)
Yu et al. (85)	c-Met	OS	HR	China	16	770^#^/1789	2.11 (1.62–2.75)
Yu et al. (86)	CRP	OS	HR	China	12	996^#^/2597	1.77 (1.56–2.00)
Zhang et al. (68)	CTCs	OS	HR	China	30	698^#^/2090	1.79 (1.49–2.15)
		RFS	HR	China	10	201^#^/781	2.91 (1.83–4.62)
Wang et al. (72)	CTCs	RFS^*^	HR	China	11	259^#^/1538	2.41 (1.93–3.01)
Liu et al. (43)	DKK1	OS	RR	China	3	209/616	2.67 (2.05–3.48)
Li et al. (78)	E-cadherin	5-year OS	RR	China	8	584^#^/1265^#^	1.61 (1.37–1.88)
Chen et al. (90)	EGFR	OS	HR	China	7	613/1289	1.66 (1.35–2.03)
Song et al. (58)	ERCC1	OS	HR	China	15	869^#^/1594	1.48 (1.02–2.13)
Guo et al. (80)	EZH2	OS	HR	China	4	282/496	1.20 (0.51–2.81)
Zeng et al. (34)	FAK	OS	HR	China	7	750^#^/2408	2.65 (1.74–4.02)
Tan et al. (87)	Fascin-1	OS	HR	UK	3	273^#^/750	1.15 (0.83–1.57)
Liu et al. (24)	FGFR2	3-year OS	OR	China	10	1154/2093	1.90 (1.17–3.07)
	FGFR2	5-year OS	OR	China	8	973/1922	1.77 (1.04–3.02)
Wang et al. (73)	FHIT	OS	HR	China	8	855^#^/1361	1.27 (1.07–1.51)
Pecqueux et al. (59)	FITC	OS	HR	Germany	51	5567^#^/11540	3.23 (2.79–3.73)
Dai et al. (66)	FOXM1	OS	HR	China	3	41^#^/220	2.27 (1.13–4.58)
Jiang et al. (26)	FOXM1	1-year OS	OR	China	6	46^#^/419	0.23 (0.11–0.48)
		3-year OS	OR	China	4	35^#^/282	0.14 (0.04–0.56)
		5-year OS	OR	China	4	38^#^/282	0.16 (0.07–0.38)
Huang et al. (97)	Foxp3+ Treg lymphocytes	1-year OS	OR	China	12	1672/1901	0.39 (0.29–0.54)
		3-year OS	OR	China	11	1167/1825	0.28 (0.21–0.38)
		5-year OS	OR	China	12	964/1888	0.31 (0.21–0.44)
Lei et al. (96)	HER2	OS	RR	China	10	2170^#^/3913	1.47 (1.09–1.98)
Gu et al. (81)	HER2	RFS	HR	China	4	701/3054	1.07 (0.84–1.37)
Cao et al. (51)	HER4	3-year OS	OR	China	3	27^#^/415	1.00 (0.85–1.18)
Zhang et al. (84)	HIF-1α	OS	HR	China	10	533/1252	1.34 (1.13–1.58)
		DFS	HR	China	5	266/403	1.67 (0.99–2.82)
Ma et al. (61)	HOTAIR	OS	HR	China	4	239/396	1.55 (0.84–2.88)
Tustumi et al. (39)	IFCC	OS	RD	Brazil	11	984^#^/2520	0.37 (0.31–0.44)
Gao et al. (63)	IGF-1R	OS	HR	China	4	373^#^/1289	2.63 (1.29–5.40)
Luo et al. (22)	Ki-67	OS	HR	China	22	1741^#^/3197	1.23 (1.06–1.42)
		DFS	HR	China	5	217/464	1.87 (1.30–2.69)
Huang et al. (46)	LGR5	OS	HR	China	4	39/359	1.66 (1.02–2.69)
Wang et al. (38)	TAMs	OS	HR	China	7	462^#^/771	1.71 (1.35–2.15)
	M2 TAM	OS	HR	China	4	537/886	1.71 (1.19–2.45)
Deng et al. (50)	MAPF	OS	HR	China	7	348^#^/871	2.74 (2.20–3.42)
		DFS	HR	China	6	381^#^/750	3.28 (1.93–5.59)
		Peritoneal RFS	HR	China	6	323/822	4.95 (3.23–7.57)
Peng et al. (76)	MET (HGFR)	OS	HR	China	16	749/2302	2.57 (1.97–3.35)
Dong et al. (64)	MMP14	OS	HR	China	3	360594	2.17 (1.64–2.86)
Shen et al. (74)	MMP2	OS	HR	China	10	1020/1514	1.92 (1.48–2.48)
Zhang et al. (91)	MMP9	OS	HR	China	11	790^#^/1611	1.25 (1.11–1.40)
Chen et al. (67)	MMP9	5-year OS	RR	China	8	328^#^/1090	1.51 (1.24–1.84)
Wang et al. (37)	MUC1	5-year OS	HR	China	4	423/758	0.28 (0.12–0.66)
Zhang et al. (52)	MUC5AC	OS	HR	China	6	422^#^/1384	1.34 (1.00–1.81)
Sun et al. (40)	NLR	OS	HR	China	19	2926^#^/5431	1.98 (1.75–2.25)
		DFS	HR	China	3	382/488	1.48 (1.05–2.09)
		PFS	HR	China	4	452/488	1.62 (1.32–1.98)
Fang et al. (29)	NM23	5-year OS	OR	China	9	732/1685	0.60 (0.24–1.46)
Han et al. (47)	NME1	OS	HR	China	5	444/960	0.75 (0.35–1.63)
Gu et al. (48)	OPN	OS	HR	China	8	879/1633	1.59 (1.15–2.22)
Wei et al. (56)	P53	OS	HR	China	21	2487^#^/4670	1.56 (1.23–1.98)
		DSS	HR	China	14	1015^#^/2053	1.59 (1.34–1.88)
Brungs et al. (33)	uPA	OS	HR	Australia	12	537^#^/1130	2.21 (1.74–2.8)
		RFS	HR	Australia	3	287/468	1.90 (1.17–1.98)
	uPAR	OS	HR	Australia	11	459^#^/1016	2.19 (1.80–2.66)
	PAI-1	OS	HR	Australia	9	407^#^/798	1.80 (1.25–2.60)
		RFS	HR	Australia	3	161/465	1.96 (1.07–3.57)
Cao et al. (32)	p-Akt	OS	HR	China	11	615^#^/1737	1.41 (1.01–1.97)
Gu et al. (27)	PD-L1	OS	HR	China	15	1312^#^/3291	1.46 (1.08–1.98)
Wu et al. (55)	PD-L1	3-year OS	OR	China	3	161/313	4.13 (1.84–9.21)
Xin-Ji et al. (53)	Platelet count	OS	HR	China	7	1132^#^/5515	1.74 (1.41–2.13)
Xu et al. (35)	PLR	OS	HR	China	7	1290^#^/4121	0.99 (0.89–1.10)
Hu et al. (89)	PRL-3	OS	HR	China	6	756^#^/1249	1.90 (1.38–2.60)
Ji et al. (45)	pSTAT3	OS	HR	China	11	815^#^/1547	1.97 (1.49–2.63)
Wang et al. (71)	S100A4	OS	HR	China	7	500^#^/866^#^	1.47 (0.77–2.81)
Jiang et al. (44)	Sirt1	3-year OS	OR	China	5	618/987	0.32 (0.19–0.55)
		5-year OS	OR	China	4	785/1264	0.44 (0.15–1.29)
Zhang et al. (69)	SK1	5-year OS	HR	China	3	597/677	1.58 (1.08–2.30)
Lin et al. (77)	SOX2	OS	HR	China	8	415/875	1.46 (0.84–2.54)
Wang et al. (70)	SPARC	OS	RR	China	6	458/851	1.67 (1.44–1.93)
Wu et al. (36)	STAT3	3-year OS	OR	China	10	960/1647	4.08 (1.81–9.21)
		5-year OS	OR	China	10	768/1647	5.47 (2.16–13.86)
Gao et al. (49)	TS	OS	HR	China	12	735^#^/2174	1.07 (0.75–1.52)
		EFS	HR	China	10	667^#^/2072	1.16 (0.84–1.61)
Chen et al. (95)	VEGF	5-year OS	RR	China	11	468^#^/1195^#^	2.43 (1.95–3.03)
Peng et al. (93)	VEGF-A	OS	HR	China	15	657^#^/2166	1.96 (1.56–2.45)
		DFS	HR	China	7	370^#^/1233	2.10 (1.57–2.81)
	VEGF-D	DFS	HR	China	5	138^#^/536	2.54 (1.58–4.07)
Cao et al. (83)	VEGF-C	OS	HR	China	11	520^#^/1594	1.67 (1.26–2.21)
		DFS	HR	China	5	217^#^/1020	1.53 (0.92–2.57)
Ge et al. (28)	VEGFR-3	3-year OS	HR	China	6	334/699	1.38 (0.93–2.04)
		5-year OS	HR	China	6	373/511	1.45 (1.06–1.97)
Chen et al. (31)	ZEB1	OS	HR	China	3	373/511	2.06 (1.49–2.84)
	ZEB2	OS	HR	China	3	309/481	2.06 (1.57–2.62)
Li et al. (79)	β-catenin	OS	HR	China	15	1215^#^/2261	1.85 (1.39–2.46)

CI, confidence interval; OS, overall survival; DFS, disease free survival; RFS, recurrence free survival; PFS, progression free survival; EFS, event-free survival; peritoneal RFS, peritoneal recurrence-free survival; DSS, disease-specifc survival; RFS^*^, relapse free survival; ARID1A, AT-rich interactive domain-containing 1A protein; BIRC5, (Survivin); PTEN, Phosphatase and tensin homolog; HIF-1α, Hypoxia inducible factor-1α; Bmi-1, B-cell-specific moloney leukemia virus insertion site 1; CA 19-9, serum carbohydrate antigen 19; CCR7, CC chemokine receptor type 7; CDH17, cadherin-17; CEA, carcinoembryonic antigen; Tissue VEGF, tissue vascular endothelial growth factor; Circulating VEGF, circulating vascular endothelial growth factor; Tissue VEGF-D, tissue vascular endothelial growth factor D; CLDN4, claudin 4; CRP, C-reactive protein; CTCs, circulating tumor cells; DKK1, Dickkopf-1; EGFR, human epidermal growth factor receptor; ERCC1, excision repair cross-complementing group 1; EZH2, Zeste homolog 2; FAK, focal adhesion kinase; FGFR2, fibroblast growth factor receptors; FHIT (bis(5′-adenosyl)-triphosphatase), fragile histidine triad protein; FITC, free intraperitoneal tumor cells; FOXM1, forkhead Box M1; HER2, human epidermal growth factor receptor-2; HOTAIR, HOX transcript antisense intergenic RNA; IFCC, intraperitoneal free cancer cell; IGF-1R, insulin-like growth factor receptor type I; LGR5, leucinerich repeat-containing G-protein-coupled receptor 5; TAMs, Tumor-associated macrophages; MAPF, molecular analysis of peritoneal fluid; MET (HGFR), hepatocyte growth factor receptor; MMP14, matrix metalloproteinase 14; MMP2, matrix metalloproteinase 2; MMP9, matrix metalloproteinase 9; MUC1, mucin 1; MUC5AC, mucin 5AC; NLR, neutrophil-to-lymphocyte ratio; NM23, non-metastatic protein 23; NME1 (NM23-H1 or NDPK-A); OPN, osteopontin; uPA, the urokinase plasminogen activation; uPAR, urokinase plasminogen activator receptor; PAI-1, plasminogen activator inhibitor-1; p-Akt, phosphorylated protein kinase B; PD-L1, programmed cell death ligand 1; PLR, platelet-lymphocyte ratio; PRL-3, phosphatase of regenerating liver 3; pSTAT3, phosphorylated signal transducer and activator of transcription proteins 3; Sirt1, silent information regulator 1; SOX2, Sex-determining region Y-box 2; SPARC (osteonectin or BM-40), secreted protein acidic and rich in cysteine; STAT3, signal transducer and activator of transcription proteins 3; TS, thymidylate synthase; VEGF, vascular endothelial growth factor; VEGF, vascular endothelial growth factor; VEGF-C, vascular endothelial growth factor-C; VEGFR-3, vascular endothelial growth factor receptors 3; ZEB1, (TCF8, AREB6 or Zfhx1a) zinc fnger E-box binding homeobox 1; ZEB2, (SIP1, HSPC082 and Zfhx1b) zinc fnger E-box binding homeobox 2.

# *Contain missing values*.

### Methodological Quality Assessment Using AMSTAR 2.0

The methodological quality of all included systematic reviews and meta-analyses was deemed critically low using the 16-item AMSTAR 2.0. Detailed results, scoring criteria, and rating criteria are shown in Supplementary Table S2. All included studies had more than two critical flaws [usually in items 2 (74/74, 100%), 7 (74/74, 100%), and 13 (74/74, 100%)] and several non-critical flaws [usually in items 3 (74/74, 100%), 10 (74/74, 100%), and 12 (74/74, 100%)]. Of note, studies with at least two critical flaws with or without non-critical flaws were considered as having critically low methodological quality.

### Summary Effect Size

The quantitative syntheses of the 120 associations were re-performed using a random-effect model to provide more conservative estimates. Forty-seven associations reached *P* < 10^−6^ (Table 2 and Supplementary Table S3). Twenty-one associations had moderate statistical significance (*P* < 10^−3^). The remaining 52 associations presented either *P* < 0.05 or no statistical significance. Most associations that reached statistical significance reported an increased risk of mortality of GC, indicating the potential prognostic effect of biomarkers for GC. Associations between Foxp3+ Treg lymphocytes and 1-, 3-, and 5-year survival of GC, between intraperitoneal free cancer cell (IFCC) and OS of GC, between Forkhead Box M1 (FOXM1) and 1- and 5-year survival of GC, between Silent information regulator 1 (Sirt1) and 3-year survival of GC, and between CC chemokine receptor type 7 (CCR7) and 5-year survival of GC all reported a decreased risk of mortality of GC.

**Table 2:** Evidence-rating results based on the results of statistical analyses of the 120 associations.

**Study**	**Association between biomarkers and gastric cancer**	Summary relative risk estimate (random-effect *P*)^*^	**Cases >1000**	**Largest studyrelative risk estimate *P* < 0.05**	***I*^**2**^ <50%**	**Smallstudy effects**	**95% prediction interval exclude the null value**	**Excess significance**	**10% credibility ceiling survival**
**Associations supported by strong evidence (1)**									
Zhang et al. (52)	platelet count OS	+++	+	+	–	–	+	–	+
**Associations supported by highly suggestive evidence (7)**									
Song et al. (57)	CA 19-9 OS	+++	+	+	–	–	–	+	+
Deng et al. (65)	CEA OS	+++	+	+	+	–	+	–	+
Pecqueux et al. (59)	FITC OS	+++	+	+	–	+	+	+	+
Huang et al. (97)	Foxp3+ Treg lymphocytes 1-year OS	+++	+	+	+	–	+	–	+
Huang et al. (97)	Foxp3+ Treg lymphocytes 3-year OS	+++	+	+	+	–	+	+	+
Sun et al. (40)	NLR OS	+++	+	+	–	–	+	+	+
Liu et al. (94)	Tissue VEGF OS	+++	+	+	–	+	–	+	+
**Associations supported by suggestive evidence (4)**									
Shen et al. (74)	MMP2 OS	+++	+	–	–	–	–	+	+
Wei et al. (56)	p53 OS	++	+	–	–	+	–	+	+
Wei et al. (56)	p53 DSS	+++	+	–	+	–	+	+	+
Li et al. (79)	β-catenin OS	++	+	–	–	+	–	–	+
**Associations supported by weak evidence (84)**									
Kim et al. (25)	ARID1A OS	++	–	+	+	–	+	+	+
Chen et al. (82)	BIRC5 5-year OS	+++	–	+	+	–	+	–	+
Shao et al. (75)	Bmi-1 OS	++	–	+	+	–	–	–	+
Song et al. (57)	CA 19-9 DFS	+	–	+	–	–	–	–	+
Song et al. (57)	CA 19-9 DSS	+	–	+	+	–	–	–	–
Du et al. (37)	CCR7 5-year OS	++	–	+	+	–	–	–	–
Lu et al. (41)	CD133 5-year OS	+++	–	+	+	+	+	–	+
Jiang et al. (26)	CD3+ T lymphocytes OS	++	–	+	+	–	–	–	+
Jiang et al. (26)	CD4+ T lymphocytes OS	+	–	+	–	–	–	–	–
Lu et al. (41)	CD44 5-year OS	+++	–	–	+	–	+	+	+
Wu et al. (54)	CD44 DFS	+	–	–	+	–	–	–	–
Jiang et al. (26)	CD8+ T lymphocytes OS	+	+	+	+	–	–	+	–
Wang et al. (92)	Cdx2 5-year OS	+++	–	+	+	–	+	–	+
Deng et al. (65)	CEA DFS	+++	–	+	+	–	+	–	+
Deng et al. (65)	CEA DSS	+++	–	+	+	–	+	–	+
Liu et al. (94)	Circulating VEGF OS	+++	–	+	+	–	–	–	+
Liu et al. (62)	CLDN4 OS	+++	–	+	+	–	+	–	+
Yu et al. (85)	c-MET OS	+++	–	–	–	–	–	+	+
Yu et al. (86)	CRP OS	+++	–	+	+	–	+	+	+
Zhang et al. (68)	CTCs OS	+++	–	+	+	–	+	–	+
Zhang et al. (68)	CTCs RFS	+++	–	+	–	–	–	–	+
Wang et al. (72)	CTCs RFS^*^	+++	–	+	+	–	+	+	+
Liu et al. (43)	DKK1 OS	+++	–	+	+	–	–	–	+
Li et al. (78)	E-cadherin 5-year OS	+++	–	+	+	–	+	–	+
Chen et al. (90)	EGFR OS	+++	–	+	+	–	+	+	+
Song et al. (58)	ERCC1 OS	+	–	+	–	–	–	+	–
Zeng et al. (34)	FAK OS	+++	–	+	–	–	–	–	+
Liu et al. (24)	FGFR2 3-year OS	+	+	+	–	–	–	–	+
Liu et al. (24)	FGFR2 5-year OS	+	–	–	–	–	–	–	–
Wang et al. (73)	FHIT OS	+	–	–	+	–	+	–	+
Dai et al. (66)	FOXM1 OS	+	–	–	+	–	–	–	–
Jiang et al. (26)	FOXM1 1-year OS	++	–	+	+	–	+	–	+
Jiang et al. (26)	FOXM1 3-year OS	+	–	+	–	–	–	–	+
Jiang et al. (26)	FOXM1 5-year OS	++	–	+	+	+	–	–	+
Huang et al. (97)	Foxp3+ Treg lymphocytes 5-year OS	+++	–	+	–	–	–	+	+
Lei et al. (96)	HER2 OS	+	+	+	–	–	–	–	–
Zhang et al. (84)	HIF-1α OS	++	–	–	+	–	+	+	+
Chen et al. (82)	HIF-1α 5-year OS	+++	–	+	+	–	+	–	+
Tustumi et al. (39)	IFCC OS	+++	–	+	+	–	+	+	+
Gao et al. (63)	IGF-1R OS	+	–	+	–	–	–	–	+
Luo et al. (22)	Ki-67 OS	+	+	–	–	+	–	+	–
Luo et al. (22)	Ki-67 DFS	++	–	–	+	–	–	+	+
Huang et al. (46)	LGR5 OS	+	–	+	–	–	–	+	–
Wang et al. (38)	M2 TAM OS	+	–	+	–	–	–	–	+
Deng et al. (50)	MAPF OS	+++	–	+	+	+	+	–	+
Deng et al. (50)	MAPF DFS	++	–	+	–	–	–	+	+
Deng et al. (50)	MAPF peritoneal RFS	+++	–	+	+	–	+	+	+
Peng et al. (76)	MET OS	+++	–	–	+	+	+	–	+
Dong et al. (64)	MMP14 OS	+++	–	+	+	–	–	–	+
Zhang et al. (91)	MMP9 OS	++	–	–	–	–	–	+	+
Chen et al. (67)	MMP9 5-year OS	++	–	+	–	+	–	–	+
Wang et al. (37)	MUC1 5-year OS	+	–	+	–	–	–	–	+
Sun et al. (40)	NLR DFS	+	–	+	+	–	+	–	–
Sun et al. (40)	NLR PFS	+++	–	+	+	–	+	+	+
Gu et al. (48)	OPN OS	+	–	+	–	–	–	+	–
Brungs et al. (33)	PAI-1 OS	+	–	+	–	–	–	+	–
Brungs et al. (33)	PAI-1 RFS	+	–	–	–	–	–	+	–
Cao et al. (32)	p-Akt OS	+	–	+	–	–	–	–	+
Gu et al. (27)	PD-L1 OS	+	+	–	–	–	–	+	–
Wu et al. (55)	PD-L1 3-year OS	++	–	+	–	–	–	–	+
Hu et al. (89)	PRL-3 OS	++	–	+	–	–	–	+	+
Ji et al. (45)	pSTAT3 OS	+++	–	+	–	–	–	–	+
Chen et al. (82)	PTEN 5-year OS	+++	–	–	+	+	+	+	+
Jiang et al. (44)	Sirt1 3-year OS	++	–	+	–	+	–	+	+
Zhang et al. (69)	SK1 5-year OS	+	–	–	+	–	–	–	–
Wang et al. (70)	SPARC OS	+++	–	+	+	–	+	+	+
Wu et al. (36)	STAT3 3-year OS	++	–	+	–	–	–	–	+
Wu et al. (36)	STAT3 5-year OS	++	–	–	–	–	–	–	+
Wang et al. (38)	TAMs OS	+++	–	–	+	–	+	+	+
Liu et al. (94)	Tissue VEGF DFS	+++	–	+	+	–	+	–	+
Liu et al. (94)	Tissue VEGF DSS	+	–	–	–	–	–	+	–
Brungs et al. (33)	uPA OS	+++	–	+	+	–	+	+	+
Brungs et al. (33)	uPA RFS	+	–	–	–	–	–	+	–
Brungs et al. (33)	uPAR OS	+++	–	+	+	+	+	–	+
Chen et al. (95)	VEGF 5-year OS	+++	–	+	–	–	+	–	+
Peng et al. (93)	VEGF-A OS	+++	–	+	+	–	+	–	+
Peng et al. (93)	VEGF-A DFS	+++	–	+	+	–	+	–	+
Cao et al. (83)	VEGF-C OS	++	–	+	+	–	–	–	+
Liu et al. (94)	VEGF-D OS	++	–	–	+	–	–	–	+
Peng et al. (93)	VEGF-D DFS	++	–	+	+	–	–	–	+
Ge et al. (28)	VEGFR-3 5–year OS	+	–	–	+	–	–	–	–
Chen et al. (31)	ZEB1 OS	+++	–	+	+	–	–	+	+
Chen et al. (31)	ZEB2 OS	+++	–	+	+	–	–	+	+
**Associations supported by not suggestive evidence (24)**									
Liu et al. (88)	BIRC5 OS	–	–	+	–	–	–	+	+
Wu et al. (54)	CD44 OS	–	–	–	+	–	–	–	–
Wu et al. (54)	CD44v6 OS	–	–	–		–	–	–	–
Lu et al. (41)	CD44v6 5-year OS	–	–	+	–	–	–	–	–
Meng et al. (60)	CDH17 5-year OS	–	–	+	–	–	–	+	–
Jiang et al. (26)	Dendritic cells OS	–	–	+	–	–	–	–	–
Guo et al. (80)	EZH2 OS	–	–	+	+	–	–	–	–
Tan et al. (87)	Fascin-1 OS	–	–	–	+	–	–	–	–
Jiang et al. (26)	Foxp3+ Treg lymphocytes OS	–	+	–	–	–	–	+	–
Gu et al. (81)	HER2 RFS	–	–	–	+	–	–	–	–
Cao et al. (51)	HER4 3-year OS	–	–	–	+	–	–	–	–
Zhang et al. (84)	HIF-1α DFS	–	–	–	–	–	–	+	–
Ma et al. (61)	HOTAIR OS	–	–	+	+	–	–	–	–
Zhang et al. (52)	MUC5AC OS	–	–	+	+	–	–	–	–
Fang et al. (29)	NM23 OS	–	–	+	–	–	–	–	–
Han et al. (47)	NME1 OS	–	–	+	–	+	+	–	–
Xu et al. (35)	PLR OS	–	+	–	+	–	–	–	–
Wang et al. (71)	S100A4 OS	–	–	–	+	+	–	+	–
Jiang et al. (44)	Sirt1 5-year OS	–	–	+	–	–	–	+	–
Lin et al. (77)	SOX2 OS	–	–	+	–	–	–	–	–
Gao et al. (49)	TS OS	–	–	–	–	–	–	–	–
Gao et al. (49)	TS EFS	–	–	–	–	–	–	–	–
Cao et al. (83)	VEGF-C DFS	–	–	–	–	–	–	–	–
Ge et al. (28)	VEGFR-3 3-year OS	–	–	–	+	–	–	–	–

* P-value calculated using random-effect model: +++P < 10^−6^; ++P < 10^−3^; +P < 0.05; –P > 0.05. For other items, + = yes, – = no. CI, confidence interval; OS, overall survival; DFS, disease free survival; RFS, recurrence free survival; PFS, progression free survival; EFS, event-free survival; peritoneal RFS, peritoneal recurrence-free survival; DSS, disease-specifc survival; RFS^^*^^, relapse free survival; ARID1A, AT-rich interactive domain-containing 1A protein; BIRC5, (Survivin); PTEN, phosphatase and tensin homolog; HIF-1α, hypoxia inducible factor-1α; Bmi-1, B-cell-specific moloney leukemia virus insertion site 1; CA 19-9, serum carbohydrate antigen 19; CCR7, CC chemokine receptor type 7; CDH17, cadherin-17; CEA, carcinoembryonic antigen; Tissue VEGF, tissue vascular endothelial growth factor; Circulating VEGF, circulating vascular endothelial growth factor; Tissue VEGF-D, tissue vascular endothelial growth factor D; CLDN4, claudin 4; CRP, C-reactive protein; CTCs, circulating tumor cells; DKK1, dickkopf-1; EGFR, human epidermal growth factor receptor; ERCC1, excision repair cross-complementing group 1; EZH2, zeste homolog 2; FAK, focal adhesion kinase; FGFR2, fibroblast growth factor receptors; FHIT (bis(5′-adenosyl)-triphosphatase), fragile histidine triad protein; FITC, free intraperitoneal tumor cells; FOXM1, forkhead box M1; HER2, human epidermal growth factor receptor-2; HOTAIR, HOX transcript antisense intergenic RNA; IFCC, intraperitoneal free cancer cell; IGF-1R, insulin-like growth factor receptor type I; LGR5, leucinerich repeat-containing G-protein-coupled receptor 5; TAMs, tumor-associated macrophages; MAPF, molecular analysis of peritoneal fluid; MET (HGFR), hepatocyte growth factor receptor; MMP14, matrix metalloproteinase 14; MMP2, matrix metalloproteinase 2; MMP9, matrix metalloproteinase 9; MUC1, mucin 1; MUC5AC, mucin 5AC; NLR, neutrophil-to-lymphocyte ratio; NM23, non-metastatic protein 23; NME1 (NM23-H1 or NDPK-A); OPN, osteopontin; uPA, the urokinase plasminogen activation; uPAR, urokinase plasminogen activator receptor; PAI-1, plasminogen activator inhibitor-1; p-Akt, phosphorylated protein kinase B; PD-L1, programmed cell death ligand 1; PLR, platelet-lymphocyte ratio; PRL-3, phosphatase of regenerating liver 3; pSTAT3, phosphorylated signal transducer and activator of transcription proteins 3; Sirt1, Silent information regulator 1; SOX2, Sex-determining region Y-box 2; SPARC (osteonectin or BM-40), secreted protein acidic and rich in cysteine; STAT3, signal transducer and activator of transcription proteins 3; TS, thymidylate synthase; VEGF, vascular endothelial growth factor; VEGF, vascular endothelial growth factor; VEGF-C, vascular endothelial growth factor-C; VEGFR-3, vascular endothelial growth factor receptors 3; ZEB1, (TCF8, AREB6 or Zfhx1a) zinc fnger E-box binding homeobox 1; ZEB2, (SIP1, HSPC082 and Zfhx1b) zinc fnger E-box binding homeobox 2.

### Heterogeneity

Seventy-six of the 120 (63.3%) associations demonstrated significant heterogeneity (*P* < 0.1), of which 54 showed high heterogeneity and 23 presented moderate to high heterogeneity. The 95% PI was also calculated to further assess inter-study heterogeneity. The 95% PIs of 38 associations excluded the null value (Table 2 and Supplementary Table S3).

### Small-Study Effects

Small study effects were found in fifteen associations: FITCs and GC OS, tissue VEGF and GC OS, β-catenin and GC OS, p53 and GC OS, MAPF and GC OS, uPAR and GC OS, MET and GC OS, CD133 and 5-year-survival of GC, PTEN and 5-year-survival of GC, FOXM1 and 5-year-survival of GC, Sirt1 and 3-year-survival of GC, MMP9 and 5-year-survival of GC, SOX2 and GC OS, S100A4 and GC OS, NME1 and GC OS all had *P* < 0.1 for Egger's test (Table 2 and Supplementary Table S3). Only one of the 120 associations contained an inadequate number of studies (<10) and failed to empower Egger's test to identify small-study effects: CD44v6 and GC OS.

## Excess Significance

Excess significance was significant (O>E and *P* < 0.1) in 45 associations (Table 2 and Supplementary Table S3).

### 10% Credibility Ceiling

Seventy-seven of the 120 associations survived the 10% credibility ceiling, including all associations graded as strong, highly suggestive, or suggestive and most of the associations classified as weak evidence. Details can be found in Table 2 and Supplementary Table S3.

### Robustness of Evidence

None of the 120 associations between prognostic biomarkers and GC survival outcomes were considered strong evidence. Only one association, namely the association between platelet count and GC OS, was supported by strong evidence. Seven associations were supported by highly suggestive evidence, including associations between free intraperitoneal tumor cells (FITCs) and GC OS, between CEA and GC OS, between neutrophils to lymphocytes ratio (NLR) and GC OS, between foxp3+ Treg lymphocytes and 1- and 3-year-OS of GC, between serum carbohydrate antigen 19-9 (CA 19-9) and GC OS, and between tissue vascular endothelial growth factor (VEGF) and GC OS (Table 2). Evidence supporting associations between p53 and OS or disease-specific survival of GC, between matrix metalloproteinase 2 (MMP2) and GC OS, and between β-catenin and GC OS were considered suggestive. The remaining 108 associations were supported by weak or not suggestive evidence. Detailed results of these analyses are shown in Supplementary Table S3.

## Discussion

### Principal Finding

Biomarkers play essential role in clinical applications during several procedures in cancers including diagnosis, treatment, and prognosis. Cancer diagnosis based on biomarkers may improve the accuracy of early diagnosis and facilitate efficient subsequent treatment. Quite a few biomarkers have been identified in clinical trials, which show promises in the benefit of cancer patients, yet limitations exist. Some appear to be predictive biomarkers and their potential of indicating cancer developments remains to be seen. Others are restricted in clinical application due to the poor efficiency of traditional detection methods such as enzyme-linked immunosorbent assay (ELISA) and polymerase chain reaction (PCR). As novel biosensing approaches sprang up, the predictive and prognostic value of the biomarker has been widely tested in clinical trials. Since clinical practitioners can hardly perform intervention in cancer patients before diagnosis, we focused more on prognostic biomarkers instead of predictive biomarkers. To evaluate the prognostic potential of existing biomarkers and to facilitate the clinical application of more robust prognostic biomarkers, we performed this umbrella review.

This umbrella review was the first to comprehensively collect existing meta-analyses and systematically appraise the robustness of evidence to provide an overview of associations between prognostic biomarkers and GC. Overall, 74 meta-analyses comprising 80 different kinds of biomarkers were included in our umbrella review, only one association (the association between platelet count and GC OS) was supported by strong evidence. Several associations were supported by highly suggestive evidence, namely associations between GC OS and free intraperitoneal tumor cells (FITC), CEA, neutrophils to lymphocytes ratio (NLR), foxp3+ Treg lymphocytes (1- and 3-year OS), serum carbohydrate antigen 19-9 (CA 19-9), and tissue vascular endothelial growth factor (VEGF). Associations between p53, matrix metalloproteinase 2 (MMP2), β-catenin and GC OS were graded as suggestive and the remaining were graded as weak evidence. These results should be interpreted cautiously considering the poor methodological quality of the included meta-analyses as ascribed by AMSTAR 2.0.

### Comparison With Other Studies and Possible Explanations

#### Classical Biomarkers and GC

CEA and CA 19-9 are two classical biomarkers detected in the last century and their predictive value for several cancers have been clinically confirmed (99, 100). However, the prognostic value of these two blood group antigens remains controversial. After systematically assessing the methodological quality and robustness of the pooled meta-analysis of 41 studies covering 14,651 participants, we found that CEA overexpression may relate to reduced OS for GC patients. However, associations between elevated CEA and GC DFS and GC disease specific survival (DSS) were found to be supported by weak evidence. These results might be explained by the low numbers of included studies and subjects: 29/3,491 for OS, 6/295 for DFS, and 7/542 for DSS. Another possible explanation is that elevated CEA is often detected in patients with GC of later stage, meaning the cause of death is not necessarily GC itself, considering severe complications. Of note, this pattern also holds for associations between CA19-9 and GC survival outcomes.

#### Novel Biomarkers and GC

Blood contains rich sources of tumor-associated biomarkers and is one of the human fluids that are easily accessible and can be analyzed in anytime and anywhere. These biomolecules are considered to be part of primary tumors, products of passive release during apoptosis and necrosis of tumor cells or biomolecules affected by tumor microenvironment (101).

In our research, we found that several biomolecules in blood may be considered as candidate prognostic biomarkers for GC patients. The association between platelet count and GC OS was the only one association that was supported by strong evidence. Platelet was previously reported to extensively interact with tumor cells, promoting tumor chemotaxis, adhesion, proliferation, and metastasis, which reasonably accounts for the robust indicative role of platelet in GC prognosis (102). High platelet count has proven to be associated with increased mortality in several cancers such as gynecologic malignancies, breast cancer, and lung cancer (103–105). Platelet count may also serve as an indicator of worse prognosis in GC based on the meta-analysis covering 5,515 subjects.

The prognosis indicative role of another inflammatory marker, NLR, is supported by highly suggestive evidence. Convincing evidence have been found between systematic inflammatory and tumor development. On one hand, myeloid growth factors secreted by cancer cells can upregulate production of neutrophils, on the other hand, immune cytokines provided by cancer cells downregulate function of lymphocyte (106). Elevated neutrophil stimulates angiogenesis and aids tumor progression while relative lymphocytopenia depresses innate anti-tumor cellular immunity, which explains why elevated NLR indicates poor OS in GC patients (107).

The other two highly suggestive evidences are that Foxp3+ Treg lymphocytes contribute to significantly poorer 1- and 3-year OS, while inconsistent result was found in 5-year OS. As a subgroup of CD4+ T help cells, Foxp3+ Treg lymphocytes play a critical role in suppressed T-cell immunity. Foxp3+ Treg lymphocytes turned out to be an unfavorable indicator of poor prognosis in GC.

Peritoneal dissemination is one of the most common and severe complications for GC. Detection of ascitic fluids and blood samples is frequently used clinically for easy accessibility and enhanced modern technologies. Evidence supporting the association between FITC and GC OS was graded as highly suggestive while the associations between circulating tumor cells (CTCs) and several GC survival outcomes were deemed to be supported by weak evidence. These results demonstrate that the role of FITC as a specific prognostic indicator of GC is more certain than that of CTC. Previous studies also suggest that FITC is a convincing predictive and prognostic biomarker for GC (108, 109) while the prognostic role of CTCs still need further confirmation.

Angiogenesis, the formation of new vascular network, plays an essential role in tumorigenesis and metastasis. As a vital target for prognosis evaluation, indicators to assess disease severity qualitatively and quantitatively are urgently needed. The vascular endothelial growth factor (VEGF) and its receptors (VEGFRs), which may modulate angiogenesis, show promises in this regard. Numerous studies report increased VEGFs and VEGFRs in both resectable and advanced GC patients. Five relevant meta-analyses of more than 11,307 participants were included in our umbrella review. The association between tissue VEGF and GC OS was supported by highly suggestive evidence while the association between tissue VEGF and GC DFS and other associations concerning VEGF, circulating VEGF, VEGF-A, VEGF-C, VEGF-D, VEGFR-3, and GC survival outcomes were supported by weak evidence. These differences can be explained by inadequate data and data quality as almost all relevant meta-analyses included less than five studies, covered fewer than 1,000 cases, or had high heterogeneity. The results concerning VEGF-C, VEGF-D are basically consistent with those concerning VEGFR-3, as the former two are essential factors in combination with the latter.

## Limitations

This umbrella review was the first to provide an overview of associations between prognostic biomarkers and GC, and several limitations exist in this work. First, the umbrella review included published meta-analyses, meaning that studies that had not been systematically evaluated were unintentionally excluded, leading to unreliable results. Second, we only focused on associations between prognostic biomarkers and GC survival outcomes, while predictive biomarkers, mostly genetic markers comprising essential component of biomarkers, were not taken into consideration. Third, the majority of cases included in these meta-analyses are from Eastern countries and in this regard, we should interpret the findings with caution when it comes to population of Western origin. Fourth, subgroup analysis was not performed due to insufficient data provided by the included meta-analyses. Future work is required to establish a more comprehensive review to assess the true associations between prognostic biomarkers and GC survival and translate these associations into clinical practice to the utmost extent.

In conclusion, the association between platelet count and GC OS was supported by strong evidence. Associations between FITC, CEA, NLR, foxp3+ Treg lymphocytes (both 1- and 3-year OS), CA 19-9, or VEGF and GC OS were supported by highly suggestive evidence, however, the results should be interpreted cautiously due to inadequate methodological quality as deemed by AMSTAR 2.0.

## Author Contributions

ZWa and XZ conceived and designed the study. CZ and XZ performed the literature search, acquired, and collated the data, which were analyzed by YS, ZWu, JSh, ZG, and JSu. ZWa was guarantor. ZWa attests that all listed authors meet authorship criteria and that no others meeting the criteria have been omitted. All authors drafted and critically revised the manuscript for important intellectual content, and gave final approval of the version to be published and contributed to the manuscript.

### Conflict of Interest

The authors declare that the research was conducted in the absence of any commercial or financial relationships that could be construed as a potential conflict of interest.
