# Supplementary material for: Prognostic Biomarkers for Gastric Cancer: An Umbrella Review of the Evidence
Source: Front Oncol. 2019 Nov 29;9:1321. doi: 10.3389/fonc.2019.01321 (PMC6895018; doi:10.3389/fonc.2019.01321)
Supplement: Supplementary file 1 [file Table_1.docx]

Supplementary Table S1: The list of excluded studies

| Study | Biomarkers | Reasons for exclusion |
| --- | --- | --- |
| Marrone et al. 2019[1] | biomarkers | did not evaluate survival outcomes |
| Szasz et al. 2016[2] | biomarkers | did not evaluate survival outcomes |
| Antonowicz et al. 2016[3] | blood test | did not evaluate survival outcomes |
| Krieg et al. 2013[4] | BIRC5 | small sample size |
| Friedenson et al. 2005[5] | BRCA1, BRCA2 | did not evaluate survival outcomes |
| Xiao et al. 2014[6] | CA 19-9 | small sample size |
| Chen et al. 2012[7] | CA724 | did not evaluate survival outcomes |
| Yiming et al. 2015[8] | CD133 | small sample size |
| Wen et al. 2013[9] | CD133 | small sample size |
| Hu et al. 2017[10] | CD147 | did not evaluate survival outcomes |
| Ni et al. 2013[11] | CD166 | included less than 3 studies |
| Hu et al, 2017[12] | CD45 RO (+) Memory T Lymphocytes | included less than 3 studies |
| Zeng et al. 2015[13] | CDH1 | did not evaluate survival outcomes |
| Tu et al. 2016[14] | CDH1 | comment |
| Long et al. 2015[15] | CDH17 | did not evaluate survival outcomes |
| Xiao et al. 2014[16] | CEA | did not evaluate survival outcomes |
| Chen et al. 2016[17] | CLDN4 | small sample size |
| Pyo et al. 2016[18] | c-MET | did not evaluate survival outcomes |
| Xie et al. 2014[19] | COL6A3 | did not evaluate survival outcomes |
| Warschkow et al. 2012[20] | CRP | did not evaluate survival outcomes |
| Arigami et al. 2017[21] | CTC | included less than 3 studies |
| Gao et al. 2013[22] | CTC | retracted article |
| Verbeke et al. 2012[23] | CXC | review |
| Han et al.2014[24] | CXCR4 | retracted article |
| Jiang et al. 2014[25] | DKK1 | review |
| Wang et al. 2015[26] | endostatin | did not evaluate survival outcomes |
| Ma et al. 2017[27] | DNMT1 | included less than 3 studies |
| Dai et al. 2017[28] | EpCAM | included less than 3 studies |
| Shen et al. 2014[29] | EphA2 | included less than 3 studies |
| Wei et al. 2014[30] | ERCC1 | small sample size |
| Wang et al. 2016[31] | EZH2 | did not evaluate survival outcomes |
| Wang et al. 2015[32] | EZH2 | small sample size |
| Liang et al. 2017[33] | Ezrin | included less than 3 studies |
| Wu et al. 2007[34] | FasL | did not find the article |
| Cao et al 2016[35] | Fbxw7 | did not evaluate survival outcomes |
| Li et al. 2017[36] | FOXM1 | small sample size |
| Liu et al. 2019[37] | GDF15 | did not evaluate survival outcomes |
| Wu et al. 2004[38] | Ghrelin | did not evaluate survival outcomes |
| Chen et al. 2017[39] | GLUT1, GLUT3 | included less than 3 studies |
| Bartley et al. 2017[40] | HER2 | did not evaluate survival outcomes |
| Wang et al. 2017[41] | HER2 | erratum |
| Creemers et al. 2017[42] | HER2 | did not evaluate survival outcomes |
| Pyo et al. 2016[43] | HER2 | did not evaluate survival outcomes |
| Zhang et al. 2015[44] | HER2 | did not evaluate survival outcomes |
| Peng et al. 2015[45] | HER2 | did not evaluate survival outcomes |
| Ye et al. 2015[46] | HER2 | small sample size |
| Liang et al. 2014[47] | HER2 | small sample size |
| Chua et al. 2012[48] | HER2 | did not evaluate survival outcomes |
| Wang et al. 2015[49] | HER2 | small sample size |
| Cao et al. 2017[50] | HER2, HER3 | included less than 3 studies |
| Ocana et al. 2013[51] | HER3 | included less than 3 studies |
| Wang et al. 2015[52] | HER3 | small sample size |
| DiVita et al. 2015[53] | HIPEC | review |
| Liu et al. 2016[54] | Hexokinase-2 | did not evaluate survival outcomes |
| Liu et al. 2016[55] | HOTAIR | did not find the article |
| Zhao et al. 2014[56] | HP CagA antibody | did not evaluate survival outcomes |
| Shiota et al. 2014[57] | HP CagA antibody | review |
| Shiota et al. 2010[58] | HP CagA antibody | did not evaluate survival outcomes |
| Azuma et al. 2004[59] | HP CagA antibody | comment |
| Huang et al.2003[60] | HP CagA antibody | did not evaluate survival outcomes |
| Ge et al. 2017[61] | HSP27 | did not evaluate survival outcomes |
| Zheng et al. 2016[62] | HIF-2α | included less than 3 studies |
| Lin et al. 2014[63] | HIF-2α | small sample size |
| Zhu et al. 2013[64] | HIF-2α | small sample size |
| Altobelli et al. 2015[65] | HtrA1 | review |
| Leake et al. 2012[66] | IFCCs | did not evaluate survival outcomes |
| Shu et al. 2015[67] | IL-6 | did not evaluate survival outcomes |
| Heatley et al. 2008[68] | Immunohistochemical biomarkers | did not evaluate survival outcomes |
| Chen et al. 2017[69] | IMP3 | included less than 3 studies |
| Pyo et al. 2017[70] | Ki-67 | small sample size |
| Wang et al. 2014[71] | LBC | did not evaluate survival outcomes |
| Chen et al. 2016[72] | LMP1 | included less than 3 studies |
| Barchitta et al. 2014[73] | Line-1 | did not evaluate survival outcomes |
| Hathurusinghe et al. 2007[74] | M2-PK | did not evaluate survival outcomes |
| Kumar et al. 2007[75] | M2-pyruvate kinase | did not evaluate survival outcomes |
| Gumulec et al. 2014[76] | Metallothionein | included less than 3 studies |
| Wang et al. 2014[77] | miRNA | did not evaluate survival outcomes |
| Wang et al. 2014[78] | MMP2 | small sample size |
| Wang et al. 2014[78] | MMP2 | small sample size |
| Long et al. 2014[79] | MMP7 | did not evaluate survival outcomes |
| Chen et al. 2014[80] | MMP9 | did not find the article |
| Sampieri et al. 2013[81] | MMPs | review |
| Tu et al. 2016[82] | MT1-MMP | letter to editor |
| Pyo et al. 2015[83] | MUC2 | did not evaluate survival outcomes |
| Yildirim et al. 2015[84] | MUC5AC | small sample size |
| Chau et al. 2004[85] | Multivariate prognostic factor | research article |
| Roli et al. 2017[86] | NGAL | included less than 3 studies |
| Hu et al. 2015[87] | NLR | review |
| Chen et al. 2015[88] | NLR | small sample size |
| Zhang et al. 2014[89] | NLR | small sample size |
| Paramanathan et al. 2014[90] | NLR | small sample size |
| Du et al. 2014[91] | Notch | review |
| Wang et al. 2014[92] | P16 | did not evaluate survival outcomes |
| Zhang et al. 2014[93] | P53 antibody | did not evaluate survival outcomes |
| Xu et al. 2014[94] | P53 | did not evaluate survival outcomes |
| Zhang et al. 2016[95] | PD-L1 | small sample size |
| Liu et al. 2016[96] | PD-L1 | small sample size |
| Xu et al. 2015[97] | PD-L1 | small sample size |
| Chong et al. 2013[98] | PI3K | research article |
| Cui et al. 2014[99] | PLCE1 | did not evaluate survival outcomes |
| Gu et al. 2016[100] | PLR | small sample size |
| Zhou et al. 2014[101] | PLR | small sample size |
| Cao et al 2016[102] | p-mTOR | review |
| Yu et al. 2016[103] | pSTAT3 | small sample size |
| Sakamoto et al. 1996[104] | preoperative immunosuppressive acidic protein | research article |
| Huang et al. 2015[105] | Serum Pepsinogens | did not evaluate survival outcomes |
| Terasewa et al. 2014[106] | Serum Pepsinogens | did not evaluate survival outcomes |
| Miki et al. 2006[107] | Serum Pepsinogens | did not evaluate survival outcomes |
| Dinis-Ribeiro et al. 2004[108] | Serum Pepsinogens | did not evaluate survival outcomes |
| Shimada et al. 2014[109] | serum tumor markers | review |
| Yu et al. 2016[110] | SIRT3 | included less than 3 studies |
| Chen et al. 2016[111] | snail protein | did not evaluate survival outcomes |
| Du et al. 2016[112] | SOX2 | small sample size |
| Chen et al. 2016[113] | SOX4 | included less than 3 studies |
| Li et al. 2016[114] | SPARC | review |
| Hu et al, 2017[115] | SPARCL1 | included less than 3 studies |
| Li et al. 2015[116] | STAT3 | did not evaluate survival outcomes |
| Liu et al. 2016[117] | TAM | did not find the article |
| Wentzensen et al. 2011[118] | telomere length | did not evaluate survival outcomes |
| Acharya et al. 2017[119] | tumor marker | did not evaluate survival outcomes |
| Li et al. 2016[120] | VacA antibody | did not evaluate survival outcomes |
| Wang et al. 2016[121] | 5-fluorouracil metabolic enzymes | small sample size |

1. Marrone MT, Tsilidis KK, Ehrhardt S, Joshu CE, Rebbeck TR, Sellers TA, Platz EA: **When Is Enough, Enough? When Are More Observational Epidemiologic Studies Needed to Resolve a Research Question: Illustrations Using Biomarker-Cancer Associations**. *Cancer epidemiology, biomarkers & prevention : a publication of the American Association for Cancer Research, cosponsored by the American Society of Preventive Oncology* 2019, **28**(2):239-247.

2. Szasz AM, Lanczky A, Nagy A, Forster S, Hark K, Green JE, Boussioutas A, Busuttil R, Szabo A, Gyorffy B: **Cross-validation of survival associated biomarkers in gastric cancer using transcriptomic data of 1,065 patients**. *Oncotarget* 2016, **7**(31):49322-49333.

3. Antonowicz S, Kumar S, Wiggins T, Markar SR, Hanna GB: **Diagnostic Metabolomic Blood Tests for Endoluminal Gastrointestinal Cancer--A Systematic Review and Assessment of Quality**. *Cancer epidemiology, biomarkers & prevention : a publication of the American Association for Cancer Research, cosponsored by the American Society of Preventive Oncology* 2016, **25**(1):6-15.

4. Krieg A, Baseras B, Tomczak M, Verde PE, Stoecklein NH, Knoefel WT: **Role of survivin as prognostic and clinicopathological marker in gastric cancer: a meta-analysis**. *Molecular biology reports* 2013, **40**(9):5501-5511.

5. Friedenson B: **BRCA1 and BRCA2 pathways and the risk of cancers other than breast or ovarian**. *MedGenMed : Medscape general medicine* 2005, **7**(2):60.

6. Xiao J, He X, Wang Z, Hu J, Sun F, Qi F, Yang S, Xiao Z: **Serum carbohydrate antigen 19-9 and prognosis of patients with gastric cancer**. *Tumour biology : the journal of the International Society for Oncodevelopmental Biology and Medicine* 2014, **35**(2):1331-1334.

7. Chen XZ, Zhang WK, Yang K, Wang LL, Liu J, Wang L, Hu JK, Zhang B, Chen ZX, Chen JP *et al*: **Correlation between serum CA724 and gastric cancer: multiple analyses based on Chinese population**. *Molecular biology reports* 2012, **39**(9):9031-9039.

8. Yiming L, Yunshan G, Bo M, Yu Z, Tao W, Gengfang L, Dexian F, Shiqian C, Jianli J, Juan T *et al*: **CD133 overexpression correlates with clinicopathological features of gastric cancer patients and its impact on survival: a systematic review and meta-analysis**. *Oncotarget* 2015, **6**(39):42019-42027.

9. Wen L, Chen XZ, Yang K, Chen ZX, Zhang B, Chen JP, Zhou ZG, Mo XM, Hu JK: **Prognostic value of cancer stem cell marker CD133 expression in gastric cancer: a systematic review**. *PloS one* 2013, **8**(3):e59154.

10. Hu C, Dong X, Wu J, Xiao F, Shang J, Liu L, Yang Y, Luo D, Li Q, Song Q *et al*: **CD147 overexpression may serve as a promising diagnostic and prognostic marker for gastric cancer: evidence from original research and literature**. *Oncotarget* 2017, **8**(19):30888-30899.

11. Ni C, Zhang Z, Zhu X, Liu Y, Qu D, Wu P, Huang J, Xu AX: **Prognostic value of CD166 expression in cancers of the digestive system: a systematic review and meta-analysis**. *PloS one* 2013, **8**(8):e70958.

12. Hu G, Wang S: **Tumor-infiltrating CD45RO(+) Memory T Lymphocytes Predict Favorable Clinical Outcome in Solid Tumors**. *Scientific reports* 2017, **7**(1):10376.

13. Zeng W, Zhu J, Shan L, Han Z, Aerxiding P, Quhai A, Zeng F, Wang Z, Li H: **The clinicopathological significance of CDH1 in gastric cancer: a meta-analysis and systematic review**. *Drug design, development and therapy* 2015, **9**:2149-2157.

14. Tu C, Yuan L, Zhou J: **Comments on a meta-analysis and systematic review of the clinicopathological significance of CDH1 in gastric cancer**. *Drug design, development and therapy* 2016, **10**:1159-1160.

15. Long ZW, Zhou ML, Fu JW, Chu XQ, Wang YN: **Association between cadherin-17 expression and pathological characteristics of gastric cancer: a meta-analysis**. *World journal of gastroenterology* 2015, **21**(12):3694-3705.

16. Xiao Y, Zhang J, He X, Ji J, Wang G: **Diagnostic values of carcinoembryonic antigen in predicting peritoneal recurrence after curative resection of gastric cancer: a meta-analysis**. *Irish journal of medical science* 2014, **183**(4):557-564.

17. Chen X, Zhao J, Li A, Gao P, Sun J, Song Y, Liu J, Chen P, Wang Z: **Clinicopathological significance of claudin 4 expression in gastric carcinoma: a systematic review and meta-analysis**. *OncoTargets and therapy* 2016, **9**:3205-3212.

18. Pyo JS, Kang G, Cho H: **Clinicopathological Significance and Diagnostic Accuracy of c-MET Expression by Immunohistochemistry in Gastric Cancer: A Meta-Analysis**. *Journal of gastric cancer* 2016, **16**(3):141-151.

19. Xie X, Liu X, Zhang Q, Yu J: **Overexpression of collagen VI alpha3 in gastric cancer**. *Oncology letters* 2014, **7**(5):1537-1543.

20. Warschkow R, Tarantino I, Ukegjini K, Beutner U, Muller SA, Schmied BM, Steffen T: **Diagnostic study and meta-analysis of C-reactive protein as a predictor of postoperative inflammatory complications after gastroesophageal cancer surgery**. *Langenbeck's archives of surgery* 2012, **397**(5):727-736.

21. Arigami T, Uenosono Y, Yanagita S, Okubo K, Kijima T, Matsushita D, Amatatsu M, Kurahara H, Maemura K, Natsugoe S: **Clinical significance of circulating tumor cells in blood from patients with gastric cancer**. *Annals of gastroenterological surgery* 2017, **1**(1):60-68.

22. Gao P, Jiao SC, Bai L, Wang H, Jing FF, Yang JL: **Detection of circulating tumour cells in gastric and hepatocellular carcinoma: a systematic review**. *The Journal of international medical research* 2013, **41**(4):923-933.

23. Verbeke H, Geboes K, Van Damme J, Struyf S: **The role of CXC chemokines in the transition of chronic inflammation to esophageal and gastric cancer**. *Biochimica et biophysica acta* 2012, **1825**(1):117-129.

24. Han M, Lv S, Zhang Y, Yi R, Huang B, Fu H, Bian R, Li X: **The prognosis and clinicopathology of CXCR4 in gastric cancer patients: a meta-analysis**. *Tumour biology : the journal of the International Society for Oncodevelopmental Biology and Medicine* 2014, **35**(5):4589-4597.

25. Jiang XT, Ma YY, Guo K, Xia YJ, Wang HJ, Li L, He XJ, Huang DS, Tao HQ: **Assessing the diagnostic value of serum Dickkopf-related protein 1 levels in cancer detection: a case-control study and meta-analysis**. *Asian Pacific journal of cancer prevention : APJCP* 2014, **15**(21):9077-9083.

26. Wang ZH, Zhu ZT, Xiao XY, Sun J: **Correlation of serum levels of endostatin with tumor stage in gastric cancer: a systematic review and meta-analysis**. *BioMed research international* 2015, **2015**:623939.

27. Ma T, Li H, Sun M, Yuan Y, Sun LP: **DNMT1 overexpression predicting gastric carcinogenesis, subsequent progression and prognosis: a meta and bioinformatic analysis**. *Oncotarget* 2017, **8**(56):96396-96408.

28. Dai M, Yuan F, Fu C, Shen G, Hu S, Shen G: **Relationship between epithelial cell adhesion molecule (EpCAM) overexpression and gastric cancer patients: A systematic review and meta-analysis**. *PloS one* 2017, **12**(4):e0175357.

29. Shen W, Xi H, Zhang K, Cui J, Li J, Wang N, Wei B, Chen L: **Prognostic role of EphA2 in various human carcinomas: a meta-analysis of 23 related studies**. *Growth factors (Chur, Switzerland)* 2014, **32**(6):247-253.

30. Wei KK, Jiang L, Wei YY, Wang YF, Qian XK, Dai Q, Guan QL: **The prognostic value of ERCC1 expression in gastric cancer patients treated with platinum-based chemotherapy: a meta-analysis**. *Tumour biology : the journal of the International Society for Oncodevelopmental Biology and Medicine* 2014, **35**(9):8721-8731.

31. Wang Y, Gao F, Zhao M, Li B, Xing D, Wang J, Yang Y: **Prognostic significance of EZH2 expression in patients with oesophageal cancer: a meta-analysis**. *Journal of cellular and molecular medicine* 2016, **20**(5):836-841.

32. Wang W, Wang F, Zong G, Liu R, Zhang Y, Luan Y, Xu L, Xuan J: **Prognostic significance of EZH2 expression in patients with digestive cancers: a meta-analysis**. *International journal of clinical and experimental medicine* 2015, **8**(9):16043-16049.

33. Liang F, Wang Y, Shi L, Zhang J: **Association of Ezrin expression with the progression and prognosis of gastrointestinal cancer: a meta-analysis**. *Oncotarget* 2017, **8**(54):93186-93195.

34. Wu W, Guan P, Yuan Y, Zhou BS: **[Meta-analysis on relationship between FasL and gastric cancer]**. *Zhonghua yu fang yi xue za zhi [Chinese journal of preventive medicine]* 2007, **41 Suppl**:25-29.

35. Cao J, Ge MH, Ling ZQ: **Fbxw7 Tumor Suppressor: A Vital Regulator Contributes to Human Tumorigenesis**. *Medicine* 2016, **95**(7):e2496.

36. Li L, Wu D, Yu Q, Li L, Wu P: **Prognostic value of FOXM1 in solid tumors: a systematic review and meta-analysis**. *Oncotarget* 2017, **8**(19):32298-32308.

37. Liu JY, Dong XX, Lu JN, Zhang Y, Liu KF, Liu LF, E QZ, Lu XJ, Yin JY, Shen YP: **Utility of GDF-15 as a diagnostic biomarker in gastric cancer: an investigation combining GEO, TCGA and meta-analysis**. *FEBS open bio* 2019, **9**(1):35-42.

38. Wu JT, Kral JG: **Ghrelin: integrative neuroendocrine peptide in health and disease**. *Annals of surgery* 2004, **239**(4):464-474.

39. Chen X, Lu P, Zhou S, Zhang L, Zhao JH, Tang JH: **Predictive value of glucose transporter-1 and glucose transporter-3 for survival of cancer patients: A meta-analysis**. *Oncotarget* 2017, **8**(8):13206-13213.

40. Bartley AN, Washington MK, Colasacco C, Ventura CB, Ismaila N, Benson AB, 3rd, Carrato A, Gulley ML, Jain D, Kakar S *et al*: **HER2 Testing and Clinical Decision Making in Gastroesophageal Adenocarcinoma: Guideline From the College of American Pathologists, American Society for Clinical Pathology, and the American Society of Clinical Oncology**. *Journal of clinical oncology : official journal of the American Society of Clinical Oncology* 2017, **35**(4):446-464.

41. **Clinicopathological factors associated with HER2-positive gastric cancer: A meta-analysis: Erratum**. *Medicine* 2017, **96**(52):e9530.

42. Creemers A, Ter Veer E, de Waal L, Lodder P, Hooijer GKJ, van Grieken NCT, Bijlsma MF, Meijer SL, van Oijen MGH, van Laarhoven HWM: **Discordance in HER2 Status in Gastro-esophageal Adenocarcinomas: A Systematic Review and Meta-analysis**. *Scientific reports* 2017, **7**(1):3135.

43. Pyo JS, Sohn JH, Kim WH: **Concordance rate between HER2 immunohistochemistry and in situ hybridization in gastric carcinoma: systematic review and meta-analysis**. *The International journal of biological markers* 2016, **31**(1):e1-10.

44. Zhang K, Cui J, Xi H, Bian S, Ma L, Shen W, Li J, Wang N, Wei B, Chen L: **Serum HER2 Is a Potential Surrogate for Tissue HER2 Status in Gastric Cancer: A Systematic Review and Meta-Analysis**. *PloS one* 2015, **10**(8):e0136322.

45. Peng Z, Zou J, Zhang X, Yang Y, Gao J, Li Y, Li Y, Shen L: **HER2 discordance between paired primary gastric cancer and metastasis: a meta-analysis**. *Chinese journal of cancer research = Chung-kuo yen cheng yen chiu* 2015, **27**(2):163-171.

46. Ye H, Chen P, Zheng Q, Wu F, Zheng C: **[Prognostic role of human epidermal growth factor receptor 2 in resectable gastric cancer: a meta-analysis]**. *Zhonghua wei chang wai ke za zhi = Chinese journal of gastrointestinal surgery* 2015, **18**(1):46-49.

47. Liang JW, Zhang JJ, Zhang T, Zheng ZC: **Clinicopathological and prognostic significance of HER2 overexpression in gastric cancer: a meta-analysis of the literature**. *Tumour biology : the journal of the International Society for Oncodevelopmental Biology and Medicine* 2014, **35**(5):4849-4858.

48. Chua TC, Merrett ND: **Clinicopathologic factors associated with HER2-positive gastric cancer and its impact on survival outcomes--a systematic review**. *International journal of cancer* 2012, **130**(12):2845-2856.

49. Wang ZQ, Sun BJ: **C-erbB-2 expression and prognosis of gastric cancer: a meta-analysis**. *Genetics and molecular research : GMR* 2015, **14**(1):1782-1787.

50. Cao GD, Chen K, Chen B, Xiong MM: **Positive prognostic value of HER2-HER3 co-expression and p-mTOR in gastric cancer patients**. *BMC cancer* 2017, **17**(1):841.

51. Ocana A, Vera-Badillo F, Seruga B, Templeton A, Pandiella A, Amir E: **HER3 overexpression and survival in solid tumors: a meta-analysis**. *Journal of the National Cancer Institute* 2013, **105**(4):266-273.

52. Wang Y, Yang H, Duan G: **HER3 over-expression and overall survival in gastrointestinal cancers**. *Oncotarget* 2015, **6**(40):42868-42878.

53. Di Vita M, Cappellani A, Piccolo G, Zanghi A, Cavallaro A, Bertola G, Bolognese A, Facchini G, D'Aniello C, Di Francia R *et al*: **The role of HIPEC in the treatment of peritoneal carcinomatosis from gastric cancer: between lights and shadows**. *Anti-cancer drugs* 2015, **26**(2):123-138.

54. Liu Y, Wu K, Shi L, Xiang F, Tao K, Wang G: **Prognostic Significance of the Metabolic Marker Hexokinase-2 in Various Solid Tumors: A Meta-Analysis**. *PloS one* 2016, **11**(11):e0166230.

55. Liu FT, Qiu C, Luo HL, Zhang Y, Xia GF, Hao TF, Zhu PQ: **The association of HOTAIR expression with clinicopathological features and prognosis in gastric cancer patients**. *Panminerva medica* 2016, **58**(2):167-174.

56. Zhao Z, Li Y, Liu S, Fu W: **Serum Helicobacter pylori CagA antibody may not be used as a tumor marker for diagnosing gastric cancer in east Asian countries**. *Tumour biology : the journal of the International Society for Oncodevelopmental Biology and Medicine* 2014, **35**(12):12217-12224.

57. Shiota S, Murakami K, Okimoto T, Kodama M, Yamaoka Y: **Serum Helicobacter pylori CagA antibody titer as a useful marker for advanced inflammation in the stomach in Japan**. *Journal of gastroenterology and hepatology* 2014, **29**(1):67-73.

58. Shiota S, Matsunari O, Watada M, Yamaoka Y: **Serum Helicobacter pylori CagA antibody as a biomarker for gastric cancer in east-Asian countries**. *Future microbiology* 2010, **5**(12):1885-1893.

59. Azuma T, Ohtani M, Yamazaki Y, Higashi H, Hatakeyama M: **Meta-analysis of the relationship between CagA seropositivity and gastric cancer**. *Gastroenterology* 2004, **126**(7):1926-1927; author reply 1927-1928.

60. Huang JQ, Zheng GF, Sumanac K, Irvine EJ, Hunt RH: **Meta-analysis of the relationship between cagA seropositivity and gastric cancer**. *Gastroenterology* 2003, **125**(6):1636-1644.

61. Ge H, He X, Guo L, Yang X: **Clinicopathological significance of HSP27 in gastric cancer: a meta-analysis**. *OncoTargets and therapy* 2017, **10**:4543-4551.

62. Zheng F, Du F, Zhao J: **Clinicopathological Differences and Prognostic Value of Hypoxia-Inducible Factor-2alpha Expression for Gastric Cancer: Evidence From Meta-Analysis**. *Medicine* 2016, **95**(7):e2871.

63. Lin S, Ma R, Zheng XY, Yu H, Liang X, Lin H, Cai XJ: **Meta-analysis of immunohistochemical expression of hypoxia inducible factor-1alpha as a prognostic role in gastric cancer**. *World journal of gastroenterology* 2014, **20**(4):1107-1113.

64. Zhu CL, Huang Q, Liu CH, Lin XS, Xie F: **Prognostic value of HIF-1alpha expression in patients with gastric cancer**. *Molecular biology reports* 2013, **40**(11):6055-6062.

65. Altobelli E, Marzioni D, Lattanzi A, Angeletti PM: **HtrA1: Its future potential as a novel biomarker for cancer**. *Oncology reports* 2015, **34**(2):555-566.

66. Leake PA, Cardoso R, Seevaratnam R, Lourenco L, Helyer L, Mahar A, Rowsell C, Coburn NG: **A systematic review of the accuracy and utility of peritoneal cytology in patients with gastric cancer**. *Gastric cancer : official journal of the International Gastric Cancer Association and the Japanese Gastric Cancer Association* 2012, **15 Suppl 1**:S27-37.

67. Shu ZB, Cao HP, Li YC, Sun LB: **Influences of laparoscopic-assisted gastrectomy and open gastrectomy on serum interleukin-6 levels in patients with gastric cancer among Asian populations: a systematic review**. *BMC gastroenterology* 2015, **15**:52.

68. Heatley MK: **Immunohistochemical biomarkers of value in distinguishing primary ovarian carcinoma from gastric carcinoma: a systematic review with statistical meta-analysis**. *Histopathology* 2008, **52**(3):267-276.

69. Chen L, Xie Y, Li X, Gu L, Gao Y, Tang L, Chen J, Zhang X: **Prognostic value of high IMP3 expression in solid tumors: a meta-analysis**. *OncoTargets and therapy* 2017, **10**:2849-2863.

70. Pyo JS, Kim NY: **Meta-analysis of prognostic role of Ki-67 labeling index in gastric carcinoma**. *The International journal of biological markers* 2017, **32**(4):e447-e453.

71. Wang L, Huang W, Du J, Chen Y, Yang J: **Diagnostic yield of the light blue crest sign in gastric intestinal metaplasia: a meta-analysis**. *PloS one* 2014, **9**(3):e92874.

72. Chen YP, Zhang WN, Chen L, Tang LL, Mao YP, Li WF, Liu X, Zhou GQ, Sun Y, Kang TB *et al*: **Effect of latent membrane protein 1 expression on overall survival in Epstein-Barr virus-associated cancers: a literature-based meta-analysis**. *Oncotarget* 2015, **6**(30):29311-29323.

73. Barchitta M, Quattrocchi A, Maugeri A, Vinciguerra M, Agodi A: **LINE-1 hypomethylation in blood and tissue samples as an epigenetic marker for cancer risk: a systematic review and meta-analysis**. *PloS one* 2014, **9**(10):e109478.

74. Hathurusinghe HR, Goonetilleke KS, Siriwardena AK: **Current status of tumor M2 pyruvate kinase (tumor M2-PK) as a biomarker of gastrointestinal malignancy**. *Annals of surgical oncology* 2007, **14**(10):2714-2720.

75. Kumar Y, Tapuria N, Kirmani N, Davidson BR: **Tumour M2-pyruvate kinase: a gastrointestinal cancer marker**. *European journal of gastroenterology & hepatology* 2007, **19**(3):265-276.

76. Gumulec J, Raudenska M, Adam V, Kizek R, Masarik M: **Metallothionein - immunohistochemical cancer biomarker: a meta-analysis**. *PloS one* 2014, **9**(1):e85346.

77. Wang R, Wen H, Xu Y, Chen Q, Luo Y, Lin Y, Luo Y, Xu A: **Circulating microRNAs as a novel class of diagnostic biomarkers in gastrointestinal tumors detection: a meta-analysis based on 42 articles**. *PloS one* 2014, **9**(11):e113401.

78. Wang HL, Zhou PY, Zhang Y, Liu P: **Relationships between abnormal MMP2 expression and prognosis in gastric cancer: a meta-analysis of cohort studies**. *Cancer biotherapy & radiopharmaceuticals* 2014, **29**(4):166-172.

79. Long ZW, Wang JL, Wang YN: **Matrix metalloproteinase-7 mRNA and protein expression in gastric carcinoma: a meta-analysis**. *Tumour biology : the journal of the International Society for Oncodevelopmental Biology and Medicine* 2014, **35**(11):11415-11426.

80. Chen J, Chen LJ, Zhou HC, Yang RB, Lu Y, Xia YL, Wu W, Hu LW: **Prognostic value of matrix metalloproteinase-9 in gastric cancer: a meta-analysis**. *Hepato-gastroenterology* 2014, **61**(130):518-524.

81. Sampieri CL, Leon-Cordoba K, Remes-Troche JM: **Matrix metalloproteinases and their tissue inhibitors in gastric cancer as molecular markers**. *Journal of cancer research and therapeutics* 2013, **9**(3):356-363.

82. Tu C, Zhou J, Yuan L: **Letter regarding "MT1-MMP is not a good prognosticator of cancer survival: evidence from 11 studies" by Wu KP et al**. *Tumour biology : the journal of the International Society for Oncodevelopmental Biology and Medicine* 2016, **37**(5):5761-5763.

83. Pyo JS, Sohn JH, Kang G, Kim DH, Kim K, Do IG, Kim DH: **MUC2 Expression Is Correlated with Tumor Differentiation and Inhibits Tumor Invasion in Gastric Carcinomas: A Systematic Review and Meta-analysis**. *Journal of pathology and translational medicine* 2015, **49**(3):249-256.

84. Yildirim M, Kaya V, Demirpence O, Gunduz S, Bozcuk H: **Prognostic significance of p53 in gastric cancer: a meta- analysis**. *Asian Pacific journal of cancer prevention : APJCP* 2015, **16**(1):327-332.

85. Chau I, Norman AR, Cunningham D, Waters JS, Oates J, Ross PJ: **Multivariate prognostic factor analysis in locally advanced and metastatic esophago-gastric cancer--pooled analysis from three multicenter, randomized, controlled trials using individual patient data**. *Journal of clinical oncology : official journal of the American Society of Clinical Oncology* 2004, **22**(12):2395-2403.

86. Roli L, Pecoraro V, Trenti T: **Can NGAL be employed as prognostic and diagnostic biomarker in human cancers? A systematic review of current evidence**. *The International journal of biological markers* 2017, **32**(1):e53-e61.

87. Hu ZD, Huang YL, Qin BD, Tang QQ, Yang M, Ma N, Fu HT, Wei TT, Zhong RQ: **Prognostic value of neutrophil to lymphocyte ratio for gastric cancer**. *Annals of translational medicine* 2015, **3**(4):50.

88. Chen J, Hong D, Zhai Y, Shen P: **Meta-analysis of associations between neutrophil-to-lymphocyte ratio and prognosis of gastric cancer**. *World journal of surgical oncology* 2015, **13**:122.

89. Zhang X, Zhang W, Feng LJ: **Prognostic significance of neutrophil lymphocyte ratio in patients with gastric cancer: a meta-analysis**. *PloS one* 2014, **9**(11):e111906.

90. Paramanathan A, Saxena A, Morris DL: **A systematic review and meta-analysis on the impact of pre-operative neutrophil lymphocyte ratio on long term outcomes after curative intent resection of solid tumours**. *Surgical oncology* 2014, **23**(1):31-39.

91. Du X, Cheng Z, Wang YH, Guo ZH, Zhang SQ, Hu JK, Zhou ZG: **Role of Notch signaling pathway in gastric cancer: a meta-analysis of the literature**. *World journal of gastroenterology* 2014, **20**(27):9191-9199.

92. Wang HL, Zhou PY, Liu P, Zhang Y: **Role of p16 gene promoter methylation in gastric carcinogenesis: a meta-analysis**. *Molecular biology reports* 2014, **41**(7):4481-4492.

93. Zhang J, Xu Z, Yu L, Chen M, Li K: **Assessment of the potential diagnostic value of serum p53 antibody for cancer: a meta-analysis**. *PloS one* 2014, **9**(6):e99255.

94. Xu HY, Xu WL, Wang LQ, Chen MB, Shen HL: **Relationship between p53 status and response to chemotherapy in patients with gastric cancer: a meta-analysis**. *PloS one* 2014, **9**(4):e95371.

95. Zhang M, Dong Y, Liu H, Wang Y, Zhao S, Xuan Q, Wang Y, Zhang Q: **The clinicopathological and prognostic significance of PD-L1 expression in gastric cancer: a meta-analysis of 10 studies with 1,901 patients**. *Scientific reports* 2016, **6**:37933.

96. Liu YX, Wang XS, Wang YF, Hu XC, Yan JQ, Zhang YL, Wang W, Yang RJ, Feng YY, Gao SG *et al*: **Prognostic significance of PD-L1 expression in patients with gastric cancer in East Asia: a meta-analysis**. *OncoTargets and therapy* 2016, **9**:2649-2654.

97. Xu F, Feng G, Zhao H, Liu F, Xu L, Wang Q, An G: **Clinicopathologic Significance and Prognostic Value of B7 Homolog 1 in Gastric Cancer: A Systematic Review and Meta-Analysis**. *Medicine* 2015, **94**(43):e1911.

98. Chong ML, Loh M, Thakkar B, Pang B, Iacopetta B, Soong R: **Phosphatidylinositol-3-kinase pathway aberrations in gastric and colorectal cancer: meta-analysis, co-occurrence and ethnic variation**. *International journal of cancer* 2014, **134**(5):1232-1238.

99. Cui XB, Peng H, Li S, Li TT, Liu CX, Zhang SM, Jin TT, Hu JM, Jiang JF, Liang WH *et al*: **Prognostic value of PLCE1 expression in upper gastrointestinal cancer: a systematic review and meta-analysis**. *Asian Pacific journal of cancer prevention : APJCP* 2014, **15**(22):9661-9666.

100. Gu X, Gao XS, Cui M, Xie M, Peng C, Bai Y, Guo W, Han L, Gu X, Xiong W: **Clinicopathological and prognostic significance of platelet to lymphocyte ratio in patients with gastric cancer**. *Oncotarget* 2016, **7**(31):49878-49887.

101. Zhou X, Du Y, Huang Z, Xu J, Qiu T, Wang J, Wang T, Zhu W, Liu P: **Prognostic value of PLR in various cancers: a meta-analysis**. *PloS one* 2014, **9**(6):e101119.

102. Cao GD, Xu XY, Zhang JW, Chen B, Xiong MM: **Phosphorylated Mammalian Target of Rapamycin p-mTOR Is a Favorable Prognostic Factor than mTOR in Gastric Cancer**. *PloS one* 2016, **11**(12):e0168085.

103. Yu S, Li G, Wang Z, Wang Z, Chen C, Cai S, He Y: **The prognostic value of pSTAT3 in gastric cancer: a meta-analysis**. *Journal of cancer research and clinical oncology* 2016, **142**(3):649-657.

104. Sakamoto J, Teramukai S, Koike A, Saji S, Ohashi Y, Nakazato H: **Prognostic value of preoperative immunosuppressive acidic protein in patients with gastric carcinoma. Findings from three independent clinical trials. Tumor Marker Committee for the Study Group of Immunochemotherapy with PSK for Gastric Cancer**. *Cancer* 1996, **77**(11):2206-2212.

105. Huang YK, Yu JC, Kang WM, Ma ZQ, Ye X, Tian SB, Yan C: **Significance of Serum Pepsinogens as a Biomarker for Gastric Cancer and Atrophic Gastritis Screening: A Systematic Review and Meta-Analysis**. *PloS one* 2015, **10**(11):e0142080.

106. Terasawa T, Nishida H, Kato K, Miyashiro I, Yoshikawa T, Takaku R, Hamashima C: **Prediction of gastric cancer development by serum pepsinogen test and Helicobacter pylori seropositivity in Eastern Asians: a systematic review and meta-analysis**. *PloS one* 2014, **9**(10):e109783.

107. Miki K: **Gastric cancer screening using the serum pepsinogen test method**. *Gastric cancer : official journal of the International Gastric Cancer Association and the Japanese Gastric Cancer Association* 2006, **9**(4):245-253.

108. Dinis-Ribeiro M, Yamaki G, Miki K, Costa-Pereira A, Matsukawa M, Kurihara M: **Meta-analysis on the validity of pepsinogen test for gastric carcinoma, dysplasia or chronic atrophic gastritis screening**. *Journal of medical screening* 2004, **11**(3):141-147.

109. Shimada H, Noie T, Ohashi M, Oba K, Takahashi Y: **Clinical significance of serum tumor markers for gastric cancer: a systematic review of literature by the Task Force of the Japanese Gastric Cancer Association**. *Gastric cancer : official journal of the International Gastric Cancer Association and the Japanese Gastric Cancer Association* 2014, **17**(1):26-33.

110. Yu FY, Xu Q, Wu DD, Lau AT, Xu YM: **The Prognostic and Clinicopathological Roles of Sirtuin-3 in Various Cancers**. *PloS one* 2016, **11**(8):e0159801.

111. Chen X, Li J, Hu L, Yang W, Lu L, Jin H, Wei Z, Yang JY, Arabnia HR, Liu JS *et al*: **The clinical significance of snail protein expression in gastric cancer: a meta-analysis**. *Human genomics* 2016, **10 Suppl 2**:22.

112. Du XM, Wang LH, Chen XW, Li YX, Li YC, Cao YW: **Prognostic value of Sox2 expression in digestive tract cancers: A meta-analysis**. *Journal of Huazhong University of Science and Technology Medical sciences = Hua zhong ke ji da xue xue bao Yi xue Ying De wen ban = Huazhong keji daxue xuebao Yixue Yingdewen ban* 2016, **36**(3):305-312.

113. Chen J, Ju HL, Yuan XY, Wang TJ, Lai BQ: **SOX4 is a potential prognostic factor in human cancers: a systematic review and meta-analysis**. *Clinical & translational oncology : official publication of the Federation of Spanish Oncology Societies and of the National Cancer Institute of Mexico* 2016, **18**(1):65-72.

114. Li Z, Li AD, Xu L, Bai DW, Hou KZ, Zheng HC, Qu XJ, Liu YP: **SPARC expression in gastric cancer predicts poor prognosis: Results from a clinical cohort, pooled analysis and GSEA assay**. *Oncotarget* 2016, **7**(43):70211-70222.

115. Hu H, Cai W, Zheng S, Ge W: **SPARCL1, a Novel Prognostic Predictive Factor for GI Malignancies: a Meta-Analysis**. *Cellular physiology and biochemistry : international journal of experimental cellular physiology, biochemistry, and pharmacology* 2017, **44**(4):1485-1496.

116. Li MX, Bi XY, Huang Z, Zhao JJ, Han Y, Li ZY, Zhang YF, Li Y, Chen X, Hu XH *et al*: **Prognostic Role of Phospho-STAT3 in Patients with Cancers of the Digestive System: A Systematic Review and Meta-Analysis**. *PloS one* 2015, **10**(5):e0127356.

117. Liu JY, Yang XJ, Geng XF, Huang CQ, Yu Y, Li Y: **Prognostic significance of tumor-associated macrophages density in gastric cancer: a systemic review and meta-analysis**. *Minerva medica* 2016, **107**(5):314-321.

118. Wentzensen IM, Mirabello L, Pfeiffer RM, Savage SA: **The association of telomere length and cancer: a meta-analysis**. *Cancer epidemiology, biomarkers & prevention : a publication of the American Association for Cancer Research, cosponsored by the American Society of Preventive Oncology* 2011, **20**(6):1238-1250.

119. Acharya A, Markar SR, Matar M, Ni M, Hanna GB: **Use of Tumor Markers in Gastrointestinal Cancers: Surgeon Perceptions and Cost-Benefit Trade-Off Analysis**. *Annals of surgical oncology* 2017, **24**(5):1165-1173.

120. Li Q, Liu J, Gong Y, Yuan Y: **Serum VacA antibody is associated with risks of peptic ulcer and gastric cancer: A meta-analysis**. *Microbial pathogenesis* 2016, **99**:220-228.

121. Wang D, Yu X, Wang X: **High/positive expression of 5-fluorouracil metabolic enzymes predicts better response to S-1 in patients with gastric cancer: a meta-analysis**. *The International journal of biological markers* 2016, **31**(2):e101-109.
