# Supplementary material for: Prognostic Biomarkers for Gastric Cancer: An Umbrella Review of the Evidence
Source: Front Oncol. 2019 Nov 29;9:1321. doi: 10.3389/fonc.2019.01321 (PMC6895018; doi:10.3389/fonc.2019.01321)
Supplement: Supplementary file 3 [file Table_3.docx]

Supplementary Table S3. Results of statistical analyses for the evidence rating of the included systematic reviews and meta-analyses

| Study | Prognosis-indicative role of a biomarker | Summary relative risk (random effect) | | Cases | Largest study | I^2^ | *P* value for Egger’s test | 95% prediction interval | Excess significance test | | 10% credibility ceiling |
| --- | --- | --- | --- | --- | --- | --- | --- | --- | --- | --- | --- |
|  |  | Estimate | *P*  value | *N* | Relative risk estimate (95% CI) |  |  |  | *O/E* | *P*  value |  |
| Kim et al, 2017 | ARID1A in OS | 1.51 (1.25-1.82) | 1.96*  10^-5^ | 344 | 1.36 (1.01-1.84) | 0% | 0.734 | (0.997-2.28) | 3/1.02 | 0.07 | 0.02 |
| Liu et al, 2013 | BIRC5 in OS | 1.15  (0.82-1.61) | 0.41 | 492 | 1.34  (1.14-1.56) | 85.20% | 0.907 | (0.28-4.67) | 9/8.19 | 0.70 | 0.006 |
| Chen et al, 2014 | BIRC5 in 5-year OS | 1.61  (1.41-1.85) | 2.40*  10^-12^ | 230 | 1.73  (1.38-2.17) | 0% | 0.452 | (1.34-1.95) | 5/3.27 | 0.16 | 0.002 |
| Shao et al, 2014 | Bmi-1 in OS | 1.50  (1.22-1.85) | 1.14*  10^-4^ | 396 | 1.42  (1.11-1.82) | 0% | 0.296 | (0.39-5.72) | 2/1.26 | 0.39 | 0.03 |
| Song et al, 2015 | CA 19-9 in OS | 1.83  (1.56-2.15) | 1.98*10^-13^ | 2609 | 1.30  (1.15-1.46) | 75.90% | 0.216 | (0.89-3.75) | 17/9.39 | 2.53*10^-3^ | 1.69*10^-6^ |
|  | CA 19-9 in DFS | 1.86  (1.17-2.96) | 9.21*10^-3^ | 497 | 1.90  (1.15-2.83) | 59.89% | →1 | (0.49-7.01) | 2/5.52 | 1.12*10^-3^ | 0.039 |
|  | CA 19-9 in DSS | 1.30  (1.04-1.61) | 0.019 | 473 | 1.47  (1.09-1.97) | 17.83% | 0.133 | (0.83-2.03) | 2/2.40 | 0.74 | 0.23 |
| Du et al, 2017 | CCR7 in 5-year OS | 0.47  (0.31-0.70) | 2.43*10^-4^ | 94 | 0.49  (0.27-0.87) | 0% | →1 | (0.19-1.14) | 3/2.36 | 0.52 | 0.53 |
| Lu et al, 2016 | CD133 in 5-year OS | 2.15  (1.71-2.70) | 5.18*10^-11^ | 901 | 1.90  (1.45-2.49) | 30.60% | 0.063 | (1.29-3.56) | 7/4.10 | 0.40 | 0.001 |
|  | CD44 in 5-year OS | 1.87  (1.55-2.66) | 8.25*10^-11^ | 653 | 1.37  (0.93-2.02) | 0% | 0.466 | (1.49-2.35) | 4/1.43 | 0.02 | 5.10*10^-4^ |
| Jiang et al, 2017 | CD3+ T lymphocytes in OS | 0.66  (0.54-0.80) | 3.78*10^-5^ | 826 | 0.65  (0.42-0.97) | 40.40% | 0.876 | (0.39-1.12) | 5/5.08 | 0.96 | 0.025 |
|  | CD4+ T lymphocytes in OS | 0.48  (0.64-1.00) | 0.0497 | 655 | 0.61  (0.44-0.85) | 52.60% | 0.175 | (0.42-1.51) | 3/5.07 | 0.16 | 0.24 |
|  | CD8+ T lymphocytes in OS | 0.83  (0.70-0.99) | 0.035 | 1012 | 0.92  (0.83-0.97) | 41.80% | 0.583 | (0.54-1.29) | 4/0.77 | 1.48*10^-4^ | 0.58 |
|  | Dendritic cells in OS | 0.62  (0.15-2.51) | 0.50 | 149 | 1.90  (1.13-3.20) | 84.50% | →1 | (0-1.47*10^7^) | 1/1.44 | 0.61 | 0.58 |
| Wu et al, 2015 | CD44 in OS | 0.91  (0.59-1.41) | 0.67 | 594 | 1.44  (0.66-2.22) | 35.8% | →1 | (0.31-2.69) | 2/1.81 | 0.87 | 0.80 |
|  | CD44 in DFS | 1.68  (1.14-2.49) | 0.009 | 121 | 1.61  (0.90-2.32) | 0% | →1 | - | 1/0.69 | 0.67 | 0.07 |
|  | CD44v6 in OS | 1.26  (0.33-4.84) | 0.74 | 154 | 1.26  (0.16-2.36) | - | - | - | 2/1.32 | 0.49 | 0.74 |
| Lu et al, 2016 | CD44v6 in 5-year OS | 1.41  (0.80-2.49) | 0.24 | 394 | 0.66  (0.49-0.89) | 82.10% | 0.462 | (0.18-10.86) | 3/1.40 | 0.11 | 0.28 |
| Meng et al,  2015 | CDH17 in 5-year OS | 0.87  (0.67-1.14) | 0.32 | 456 | 0.78  (0.69-0.89) | 91.60% | →1 | (0.35-2.19) | 5/2.83 | 0.076 | 0.92 |
| Wang et al,  2012 | Cdx2 in 5-year OS | 2.21  (1.78-2.74) | 7.08*10^-13^ | 199 | 2.27  (1.55-3.32) | 0% | 0.308 | (1.37-3.55) | 4/2.63 | 0.15 | 0.011 |
| Deng et al,  2015 | CEA in OS | 1.73  (1.57-1.90) | 1.62*10^-28^ | 3491 | 1.61  (1.36-1.89) | 29.20% | 0.806 | (1.29-2.31) | 17/14.69 | 0.39 | 8.16*10^-7^ |
|  | CEA in DFS | 2.27  (1.71-3.01) | 1.49*10^-8^ | 295 | 2.24  (1.56-3.22) | 34.70% | 0.707 | (1.13-4.54) | 4/4.04 | 0.97 | 0.012 |
|  | CEA in DSS | 1.95  (1.50-2.54) | 7.05*10^-7^ | 542 | 1.72  (1.09-2.70) | 29.70% | →1 | (1.07-3.57) | 4/2.85 | 0.38 | 3.79*10^-3^ |
| Liu et al,  2012 | Circulating VEGF in OS | 4.22  (2.47-7.18) | 1.21*10^-7^ | 105 | 4.04  (2.08-7.84) | 0% | 0.296 | (0.13-133.53) | 3/2.31 | 0.34 | 0.028 |
| Liu et al,  2015 | CLDN4 in OS | 2.01  (1.62-2.50) | 2.21*10^-10^ | 378 | 2.07  (1.97-4.03) | 0% | →1 | (1.52-2.67) | 5/5.17 | 0.88 | 0.009 |
| Yu et al,  2013 1 | c-MET in OS | 2.11  (1.62-2.75) | 2.54*10^-8^ | 770 | 1.34  (0.93-1.92) | 52.30% | 0.26 | (0.93-4.81) | 11/6.52 | 0.023 | 4.86*10^-4^ |
| Yu et al,  2013 2 | CRP in OS | 1.77  (1.56-2.00) | 3.94*10^-19^ | 996 | 1.54  (1.25-1.92) | 18.90% | 0.193 | (1.38-2.27) | 9/4.25 | 0.004 | 4.10*10^-4^ |
| Zhang et al,  2014 | CTCs in OS | 1.79  (1.49-2.15) | 5.19*10^-10^ | 698 | 1.34  (1.14-1.56) | 30.70% | 0.225 | (1.06-3.01) | 11/8.21 | 0.25 | 3.81*10^-5^ |
|  | CTCs in RFS | 2.91  (1.83-4.62) | 6.27*10^-6^ | 201 | 3.46  (2.12-5.64) | 52.90% | 0.721 | (0.83-10.22) | 7/6.00 | 0.52 | 0.012 |
| Wang et al,  2014 | CTCs in Relapse FS | 2.41  (1.93-3.01) | 6.59*10^-15^ | 259 | 2.34  (1.48-3.70) | 0% | 0.119 | (1.87-3.11) | 9/4.52 | 0.006 | 7.17*10^-4^ |
| Liu et al,  2016 | DKK1 in OS | 2.67  (2.05-3.48) | 3.90*10^-13^ | 209 | 2.64  (1.98-3.51) | 0% | →1 | (0.48-14.85) | 3/1.97 | 0.21 | 0.027 |
| Chen et al,  2013 | EGFR in OS | 1.66  (1.35-2.03) | 8.57*10^-7^ | 613 | 1.49  (1.10-2.13) | 0% | →1 | (1.27-2.16) | 4/1.55 | 0.026 | 0.003 |
| Song et al,  2015 | ERCC1 in OS | 1.48  (1.02-2.13) | 0.036 | 869 | 1.37  (1.08-1.75) | 83.80% | 0.276 | (0.35-6.25) | 10/2.52 | 2.39*10^-7^ | 0.11 |
| Guo et al,  2014 | EZH2 in OS | 1.20  (0.51-2.81) | 0.68 | 282 | 2.02  (1.00-4.11) | 46.1% | 0.308 | (0.06-25.89) | 1/2.01 | 0.31 | 0.37 |
| Zeng et al,  2012 | FAK in OS | 2.65  (1.74-4.02) | 4.92*10^-6^ | 750 | 1.71  (1.32-2.23) | 74.90% | 0.548 | (0.73-9.53) | 6/4.46 | 0.23 | 0.008 |
| Tan et al,  2013 | Fascin-1 in OS | 1.15  (0.83-1.57) | 0.399 | 273 | 1.33  (0.90-1.96) | 8.50% | 0.296 | (0.11-11.86) | 0/2.42 | 4.03*10^-4^ | 0.45 |
| Liu et al,  2017 | FGFR2 in 3-year OS | 1.90  (1.17-3.07) | 0.009 | 1154 | 2.43  (1.19-4.95) | 55.80% | →1 | (0.46-7.80) | 3/8.27 | 1.05*10^-5^ | 0.031 |
|  | FGFR2 in 5-year OS | 1.77  (1.04-3.02) | 0.036 | 973 | 0.53  (0.25-1.11) | 53.80% | 0.711 | (0.39-7.99) | 2/5.57 | 0.006 | 0.081 |
| Wang et al,  2014 | FHIT in OS | 1.27  (1.07-1.51) | 0.007 | 855 | 1.28  (0.96-1.71) | 0% | 0.902 | (1.02-1.58) | 0/2.66 | 0.046 | 0.027 |
| Pecqueux et al, 2015 | FITC in OS | 3.23  (2.79-3.73) | →0 | 5567 | 1.34  (1.12-1.59) | 73.90% | 0.001 | (1.39-7.49) | 50/14.29 | 8.53*10^-29^ | 4.73*10^-15^ |
| Dai et al, 2015 | FOXM1 in OS | 2.27  (1.13-4.58) | 0.022 | 41 | 1.98  (0.84-4.66) | 0% | →1 | (0.02-214.67) | 1/1.53 | 0.54 | 0.066 |
| Jiang et al, 2017 | FOXM1 in 1-year OS | 0.23  (0.11-0.48) | 1.28*10^-4^ | 46 | 0.23  (0..06-0.86) | 0% | →1 | (0.08-0.66) | 2/4.13 | 0.06 | 0.019 |
|  | FOXM1 in 3-year OS | 0.14  (0.04-0.56) | 0.005 | 35 | 0.29  (0.10-0.85) | 77.30% | 0.734 | (0-59.55) | 3/3.27 | 0.73 | 0.017 |
|  | FOXM1 in 5-year OS | 0.16  (0.07-0.38) | 2.91*10^-5^ | 38 | 0.30  (0.11-0.81) | 37% | 0.089 | (0.01-3.08) | 4/3.26 | 0.34 | 0.015 |
|  | Foxp3+ Treg lymphocytes in OS | 0.97  (0.74-1.28) | 0.84 | 1147 | 0.83  (0.58-1.12) | 73.40% | 0.721 | (0.31-3.02) | 11/1.77 | 3.68*10^-13^ | 0.60 |
| Huang et al, 2014 | Foxp3+ Treg lymphocytes in 1-year OS | 0.39  (0.29-0.54) | 6.79*10^-9^ | 1672 | 0.31  (0.14-0.68) | 1.50% | 0.732 | (0.27-0.58) | 8/8.03 | 0.99 | 0.002 |
|  | Foxp3+ Treg lymphocytes in 3-year OS | 0.28  (0.21-0.38) | 5.65*10^-18^ | 1167 | 0.53  (0.33-0.85) | 38.30% | 0.876 | (0.14-0.59) | 10/5.71 | 0.01 | 6.40*10^-5^ |
|  | Foxp3+ Treg lymphocytes in 5-year OS | 0.31  (0.21-0.44) | 2.02*10^-10^ | 964 | 0.25  (0.16-0.39) | 78.30% | 0.764 | (0.009-1.01) | 10/11.21 | 0.016 | 0.004 |
| Lei et al, 2017 | HER2 in OS | 1.47  (1.09-1.98) | 0.01 | 2170 | 1.96  (1.51-2.55) | 69.30% | →1 | (0.57-3.81) | 4/6.96 | 0.04 | 0.29 |
| Gu et al, 2014 | HER2 in Relapse-FS | 1.07  (0.84-1.37) | 0.56 | 701 | 0.99  (0.72-1.37) | 0% | 0.734 | (0.63-1.83) | 0/0.20 | 0.65 | 0.56 |
| Cao et al, 2016 | HER4 in 3-year OS | 1.00  (0.85-1.18) | 0.97 | 27 | 0.89  (0.71-1.12) | 8.80% | 0.296 | (0.30-3.36) | 0/2.06 | 0.01 | 0.97 |
| Zhang et al, 2013 | HIF-1α in OS | 1.34  (1.13-1.58) | 8.23*10^-4^ | 533 | 1.11  (0.84-1.45) | 0% | 0.858 | (1.09-1.63) | 2/0.61 | 0.07 | 0.03 |
|  | HIF-1α in DFS | 1.67  (0.99-2.82) | 0.056 | 266 | 1.00  (0.69-1.45) | 62.00% | 0.462 | (0.33-8.51) | 2/0.25 | 3.30*10^-4^ | 0.33 |
| Chen et al, 2014 | HIF-1α in 5-year OS | 1.52  (1.28-1.81) | 1.73*10^-6^ | 454 | 1.56  (1.17-2.09) | 34.70% | 0.371 | (1.00-2.32) | 4/3.94 | 0.97 | 0.005 |
| Liu et al, 2016 | HK-2 in OS | 1.90  (1.51-2.38) | 3.62*10^-8^ | 1781 | 2.01  (1.36-3.00) | 55.40% | 0.544 | (0.88-4.09) | 11/10.85 | 0.94 | 1.32*10^-4^ |
| Ma et al, 2015 | HOTAIR in OS | 1.55  (0.84-2.88) | 0.16 | 239 | 2.83  (1.35-5.92) | 36.10% | 0.734 | (0.19-12.95) | 1/3.07 | 0..014 | 0.43 |
| Tustumi et al, 2016 | IFCC in OS | 0.37  (0.31-0.44) | 3.91*10^-29^ | 984 | 0.48  (0.39-0.58) | 40.80% | 0.592 | (0.24-0.57) | 11/8.09 | 0.047 | 7.76*10^-5^ |
| Gao et al, 2015 | IGF-1R in OS | 2.63  (1.29-5.40) | 0.008 | 373 | 5.31  (3.47-8.13) | 81.60% | 0.734 | (0.11-65.29) | 2/3.99 | 2.47*10^-88^ | 0.047 |
| Luo et al,  2017 | Ki-67 in OS | 1.23  (1.06-1.42) | 0.005 | 1721 | 1.02  (0.99-1.05) | 79.80% | 0.061 | (0.69-2.18) | 7/3.01 | 0.013 | 0.19 |
|  | Ki-67 in DFS | 1.87  (1.30-2.69) | 7.99*10^-4^ | 217 | 1.35  (0.78-2.35) | 31.00% | 0.221 | (0.73-4.81) | 2/0.58 | 0.047 | 0.02 |
| Huang et al,  2016 | LGR5 in OS | 1.66  (1.02-2.70) | 0.041 | 39 | 1.66  (1.22-2.25) | 70.20% | 0.308 | (0.23-11.92) | 2/0.48 | 0.019 | 0.26 |
| Wang et al,  2016 | M2 TAM in OS | 1.71  (1.19-2.45) | 0.004 | 537 | 2.30  (2.17-2.44) | 77.10% | 0.734 | (0.39-7.51) | 2/3.05 | 0.22 | 0.049 |
| Deng et al,  2016 | MAPF in OS | 2.74  (2.20-3.42) | 4.16*10^-19^ | 348 | 2.58  (1.88-3.52) | 0% | 0.035 | (2.05-3.67) | 7/5.76 | 0.22 | 0.001 |
|  | MAPF in DFS | 3.28  (1.93-5.59) | 1.16*10^-5^ | 381 | 2.36  (1.62-3.45) | 52.30% | 0.133 | (0.80-13.41) | 6/3.39 | 0.003 | 0.008 |
|  | MAPF in peritoneal RFS | 4.95  (3.23-7.57) | 1.69*10^-13^ | 323 | 3.20  (1.55-6.62) | 7.30% | 0.452 | (2.39-10.22) | 6/4.47 | 0.15 | 0.003 |
| Peng et al,  2014 | MET in OS | 2.57  (1.97-3.35) | 4.49*10^-12^ | 749 | 1.34  (0.94-1.92) | 49.70% | 0.043 | (1.14-5.76) | 12/2.02 | 5.82*10^-14^ | 3.19*10^-5^ |
| Dong et al,  2015 | MMP14 in OS | 2.17  (1.64-1.86) | 4.43*10^-8^ | 360 | 1.86  (1.23-2.80) | 0% | 0.296 | (0.36-13.07) | 3/1.88 | 0.18 | 0.03 |
| Shen et al,  2014 | MMP2 in OS | 1.92  (1.48-2.48) | 6.09*10^-7^ | 1020 | 1.21  (0.90-1.65) | 62.60% | 0.721 | (0.87-4.24) | 6/0.92 | 2.73*10^-8^ | 0.002 |
| Zhang et al,  2012 | MMP9 in OS | 1.25  (1.11-1.40) | 1.41*10^-4^ | 790 | 1.06  (0.96-1.18) | 66.10% | 0.276 | (0.88-1.77) | 6/3.43 | 0.09 | 0.009 |
| Chen et al,  2015 | MMP9 in 5-year OS | 1.51  (1.24-1.84) | 4.65*10^-5^ | 328 | 1.26  (1.07-1.48) | 58.50% | 0.063 | (0.86-2.67) | 4/5.25 | 0.35 | 0.007 |
| Wang et al,  2016 | MUC1 in 5-year OS | 0.28  (0.12-0.66) | 0.004 | 423 | 0.49  (0.28-0.86) | 81.80% | 0.734 | (0.01-13.25) | 3/2.82 | 0.87 | 0.0497 |
| Zhang et al,  2015 | MUC5AC in OS | 1.34  (1.00-1.81) | 0.051 | 422 | 1.45  (1.00-2.10) | 31.50% | 0.707 | (0.66-2.73) | 2/2.43 | 0.72 | 0.15 |
| Sun et al,  2016 | NLR in OS | 1.98  (1.75-2.25) | 6.07*10^-27^ | 2926 | 2.14  (1.98-2.31) | 53.60% | 0.162 | (1.37-2.88) | 16/10.36 | 0.009 | 2.26*10^-7^ |
|  | NLR in DFS | 1.48  (1.05-2.09) | 0.02 | 382 | 1.65  (1.09-2.49) | 0% | 0.296 | (0.16-13.73) | 1/0.61 | 0.58 | 0.24 |
|  | NLR in PFS | 1.62  (1.32-1.98) | 4.70*10^-6^ | 452 | 1.48  (1.14-1.91) | 0% | 0.734 | (1.03-2.54) | 3/0.35 | 2.74*10^-6^ | 0.03 |
| Fang et al,  2017 | NM23 in 5-year OS | 0.60  (0.24-1.46) | 0.26 | 732 | 2.16  (1.50-3.13) | 89.50% | 0.711 | (0.03-12.72) | 5/4.88 | 0.94 | 0.54 |
| Han et al,  2016 | NME1 in OS | 0.75  (0.35-1.63) | 0.47 | 444 | 2.08  (1.21-3.58) | 79.10% | 0.086 | (0.05-11.48) | 1/3.15 | 0.046 | 0.83 |
| Gu et al,  2016 | OPN in OS | 1.59  (1.15-2.22) | 0.006 | 879 | 0.74  (0.55-0.99) | 80.60% | 0.174 | (0.53-4.81) | 6/1.69 | 1.89*10^-4^ | 0.07 |
| Wei et al,  2015 | P53 in OS | 1.56  (1.23-1.98) | 2.92*10^-4^ | 2487 | 1.11  (0.95-1.31) | 85.70% | 0.085 | (0.54-4.51) | 11/4.26 | 2.55*10^-4^ | 0.002 |
|  | P53 in DSS | 1.59  (1.34-1.88) | 1.01*10^-7^ | 1015 | 1.26  (1.00-1.59) | 34.20% | 0.228 | (1.03-2.45) | 8/3.33 | 0.003 | 8.66*10^-4^ |
| Brungs et al,  2017 | PAI-1 in OS | 1.80  (1.25-2.60) | 0.002 | 407 | 1.62  (1.18-2.21) | 65.70% | 0.348 | (0.60-5.45) | 5/2.71 | 0.096 | 0.84 |
|  | PAI-1 in RFS | 1.96  (1.08-3.57) | 0..03 | 161 | 1.19  (0.82-1.72) | 75.90% | 0.296 | (0-2212.4) | 2/0.26 | 3.56*10^-4^ | 0.14 |
| Cao et al,  2017 | p-Akt in OS | 1.41  (1.01-1.97) | 0.04 | 615 | 1.93  (1.38-2.70) | 61.70% | 0.138 | (0.52-3.80) | 4/5.87 | 0.26 | 0.01 |
| Gu et al,  2017 | PD-L1 in OS | 1.46  (1.08-1.98) | 0.01 | 1312 | 0.88  (0.62-1.25) | 78.90% | 0.276 | (0.46-4.61) | 7/2.03 | 1.76*10^-4^ | 0.47 |
| Wu et al,  2015 | PD-L1 in 3-year OS | 4.13  (1.84-9.25) | 5.84*10^-4^ | 161 | 8.15  (3.30-10.14) | 72.30% | →1 | (0-45169.5) | 3/3.00 | 0.99 | 0.03 |
| Zhang et al,  2015 | platelet count in OS | 1.74  (1.41-2.13) | 1.61*10^-7^ | 1132 | 1.54  (1.17-2.11) | 37.70% | →1 | (1.05-2.87) | 6/5.35 | 0.56 | 0.007 |
| Xu et al,  2016 | PLR in OS | 0.99  (0.89-1.10) | 0.85 | 1290 | 1.19  (0.96-1.48) | 11.00% | 0.368 | (0.82-1.19) | 0/1.96 | 0.10 | 0.77 |
| Hu et al,  2013 | PRL-3 in OS | 1.90  (1.38-2.60) | 7.02*10^-5^ | 756 | 1.24  (1.02-1.49) | 69.00% | →1 | (0.73-4.94) | 4/1.70 | 0.04 | 0.009 |
| Ji et al,  2016 | pSTAT3 in OS | 1.97  (1.49-2.63) | 2.78*10^-6^ | 815 | 2.10  (1.53-2.89) | 61.70% | 0.35 | (0.82-4.76) | 6/6.41 | 0.80 | 0.008 |
| Chen et al,  2014 | PTEN in 5-year OS | 1.59  (1.38-1.84) | 2.68*10^-10^ | 639 | 1.20  (0.95-1.51) | 36.10% | 0.029 | (1.12-2.27) | 8/2.67 | 0.0001 | 8.15*10^-4^ |
| Wang et al,  2014 | S100A4 in OS | 1.47  (0.77-2.81) | 0.24 | 500 | 1.40  (0.46-4.29) | 0% | 0.009 | (0.59-3.69) | 0/2.12 | 0.08 | 0.24 |
| Jiang et al,  2016 | Sirt1 in 3-year OS | 0.32  (0.19-0.55) | 4.17*10^-5^ | 618 | 0.58  (0.39-0.87) | 57.90% | 0.086 | (0.06-1.77) | 5/2.16 | 0.01 | 0.01 |
|  | Sirt1 in 5-year OS | 0.44  (0.15-1.29) | 0.13 | 785 | 0.61  (0.41-0.90) | 92.10% | 0.734 | (0-64.43) | 4/2.24 | 0.08 | 0.49 |
| Zhang et al,  2014 | SK1 in 5-year OS | 1.58  (1.08-2.30) | 0.02 | 597 | 1.36  (0.85-2.18) | 0% | →1 | (0.14-18.41) | 1/0.38 | 0.28 | 0..053 |
| Lin et al,  2014 | SOX2 in OS | 1.46  (0.84-2.54) | 0.18 | 415 | 3.12  (1.81-5.37) | 75.20% | 0.063 | (0.25-8.69) | 3/5.56 | 0.049 | 0.23 |
| Wang et al,  2014 | SPARC in OS | 1.67  (1.44-1.93) | 5.42*10^-12^ | 458 | 1.41  (1.09-1.82) | 0% | 0.452 | (1.36-2.05) | 4/1.16 | 0.003 | 0.003 |
| Wu et al,  2016 | STAT3 3-year OS | 4.08  (1.81-9.21) | 7.12*10^-4^ | 960 | 0.42  (0.27-0.74) | 89.90% | 0.152 | (0.21-78.43) | 8/7.11 | 0.53 | 0.02 |
|  | STAT3 5-year OS | 5.47  (2.16-13.86) | 3.48*10^-4^ | 768 | 1.63  (0.96-2.78) | 90.20% | 0.371 | (0.2-149.47) | 8/3.52 | 0.003 | 0.04 |
| Wang et al,  2016 | TAMs in OS | 1.71  (1.35-2.15) | 6.44*10^-6^ | 462 | 1.41  (0.93-2.13) | 17.50% | →1 | (1.08-2.69) | 4/1.76 | 0.051 | 0.01 |
| Liu et al,  2012 | Tissue VEGF in OS | 2.13  (1.71-2.64) | 8.43*10^-12^ | 1056 | 1.44  (1.05-1.99) | 53.20% | 0.043 | (0.99-4.58) | 15/4.57 | 3.47*10^-8^ | 1.10*10^-4^ |
|  | Tissue VEGF in DFS | 2.03  (1.57-2.62) | 6.04*10^-8^ | 465 | 2.19  (1.39-3.44) | 6.70% | 0.368 | (1.35-3.05) | 4/4.47 | 0.71 | 0.013 |
|  | Tissue VEGF in DSS | 2.59  (1.33-5.06) | 0.005 | 190 | 1.52  (0.94-2.46) | 65.90% | 0.296 | (0-4270.59) | 2/0.75 | 0.096 | 0.06 |
| Gao et al,  2016 | TS in OS | 1.07  (0.75-1.52) | 0.71 | 735 | 0.87  (0.59-1.27) | 76.60% | 0.115 | (0.31-3.63) | 7/4.66 | 0.17 | 0.76 |
|  | TS in EFS | 1.16  (0.84-1.61) | 0.37 | 667 | 1.11  (0.88-1.41) | 68.70% | 0.371 | (0.43-3.11) | 4/4.45 | 0.77 | 0.59 |
| Brungs et al,  2017 | uPA in OS | 2.21  (1.74-2.80) | 6.46*10^-11^ | 557 | 1.92  (1.20-3.07) | 30.90% | 0.15 | (1.24-3.93) | 9/5.71 | 0.06 | 5.85*10^-4^ |
|  | uPA in RFS | 1.90  (1.17-3.10) | 0.01 | 287 | 1.30  (0.89-1.88) | 59.30% | 0.296 | (0.01-374.27) | 2/0.41 | 0.008 | 0.06 |
|  | uPAR in OS | 2.19  (1.80-2.66) | 2.84*10^-15^ | 459 | 2.20  (1.40-3.46) | 0% | 0.043 | (1.75-2.75) | 8/5.52 | 0.13 | 3.74*10^-5^ |
| Chen et al,  2012 | VEGF in 5-year OS | 2.43  (1.95-3.03) | 3.52*10^-15^ | 468 | 2.68  (1.94-3.70) | 50.70% | 0.533 | (1.29-4.57) | 9/7.80 | 0.43 | 1.77*10^-4^ |
| Peng et al,  2012 | VEGF-A in OS | 1.96  (1.56-2.45) | 4.73*10^-9^ | 657 | 1.75  (1.25-2.46) | 39.70% | 0.621 | (1.05-3.65) | 8/9.00 | 0.60 | 0.001 |
|  | VEGF-A in DFS | 2.10  (1.57-2.81) | 5.04*10^-7^ | 370 | 2.19  (1.12-2.77) | 24.90% | →1 | (1.12-3.94) | 4/4.94 | 0.44 | 0.009 |
| Cao et al,  2014 | VEGF-C in OS | 1.67  (1.26-2.21) | 3.68*10^-4^ | 520 | 2.58  (1.71-3.90) | 49.20% | 0.213 | (0.76-3.67) | 4/8.10 | 0.005 | 0.036 |
|  | VEGF-C in DFS | 1.53  (0.92-2.57) | 0.10 | 217 | 1.16  (0.76-1.79) | 73.10% | →1 | (0.26-9.13) | 1/3.20 | 0.04 | 0.17 |
| Liu et al,  2012 | VEGF-D in OS | 1.73  (1.25-2.40) | 9.94*10^-4^ | 99 | 1.60  (0.99-2.57) | 0% | →1 | (0.85-3.55) | 2/1.50 | 0.61 | 0.04 |
| Peng et al,  2012 | VEGF-D in DFS | 2.54  (1.58-4.07) | 1.12*10^-4^ | 138 | 4.49  (2.42-8.30) | 44.10% | 0.806 | (0.65-9.99) | 3/4.95 | 1.87*10^-18^ | 0.04 |
| Ge et al,  2017 | VEGFR-3 in 3-year OS | 1.38  (0.93-2.04) | 0.11 | 334 | 1.44  (0.83-2.49) | 0% | →1 | (0.79-2.40) | 0/1.10 | 0.25 | 0.17 |
|  | VEGFR-3 in 5-year OS | 1.45  (1.06-1.97) | 0.02 | 443 | 1.45  (0.87-2.39) | 5.60% | 0.452 | (0.87-2.41) | 1/1.04 | 0.97 | 0.17 |
| Chen et al,  2017 | ZEB1 in OS | 2.06  (1.49-2.84) | 9.92*10^-6^ | 373 | 1.71  (1.23-2.37) | 27.90% | →1 | (0.12-35.78) | 3/1.05 | 0.02 | 0.03 |
|  | ZEB2 in OS | 2.06  (1.58-2.69) | 9.77*10^-8^ | 309 | 1.93  (1.27-2.56) | 0% | 0.296 | (0.37-11.56) | 3/1.37 | 0.06 | 0.03 |
| Li et al,  2014 | β-catenin in OS | 1.85  (1.39-2.46) | 2.30*10^-5^ | 1215 | 0.74  (0.54-1.01) | 70.60% | 0.092 | (0.69-4.96) | 8/5.62 | 0.20 | 0.006 |

-: Not available

→1: Infinitely close to 1

Abbreviations: CI: confidence interval; OS: overall survival; DFS: disease free survival; RFS: recurrence free survival; PFS: progression free survival; EFS: event-free survival; peritoneal RFS: peritoneal recurrence-free survival; DSS: disease-specifc survival ; RFS^*^: relapse free survival ; ARID1A: AT‑rich interactive domain‑containing 1A protein; BIRC5: (Survivin); PTEN: Phosphatase and tensin homolog; HIF-1α: Hypoxia inducible factor-1α; Bmi-1: B-cell-specific moloney leukemia virus insertion site 1; CA 19-9: Serum Carbohydrate Antigen 19; CCR7: CC chemokine receptor type 7; CDH17: Cadherin-17; CEA: Carcinoembryonic antigen; Tissue VEGF: Tissue Vascular endothelial growth factor; Circulating VEGF: Circulating Vascular endothelial growth factor; Tissue VEGF-D: Tissue Vascular endothelial growth factor D; CLDN4: Claudin 4; CRP: C-reactive Protein; CTCs: Circulating tumor cells; DKK1: Dickkopf-1; EGFR: Human Epidermal Growth Factor Receptor; ERCC1: Excision repair cross-complementing group 1; EZH2: Zeste homolog 2; FAK: Focal Adhesion Kinase; FGFR2: Fibroblast growth factor receptors; FHIT (bis(5′-adenosyl)-triphosphatase): Fragile histidine triad protein; FITC: Free intraperitoneal tumor cells; FOXM1: Forkhead Box M1; HER2: Human epidermal growth factor receptor-2; HOTAIR: HOX transcript antisense intergenic RNA; IFCC: Intraperitoneal free cancer cell; IGF-1R: Insulin-like growth factor receptor type I; LGR5: Leucinerich repeat-containing G-protein-coupled receptor 5; TAMs: Tumor-associated macrophages; MAPF: Molecular analysis of peritoneal fluid; MET (HGFR): Hepatocyte growth factor receptor; MMP14: Matrix metalloproteinase 14; MMP2: Matrix metalloproteinase 2; MMP9: Matrix metalloproteinase 9; MUC1: Mucin 1; MUC5AC: Mucin 5AC; NLR : Neutrophil-to-lymphocyte ratio; NM23: Nonmetastatic protein 23; NME1 (NM23-H1 or NDPK-A); OPN: Osteopontin; uPA: The urokinase plasminogen activation; uPAR : Urokinase plasminogen activator receptor; PAI-1: Plasminogen activator inhibitor-1; p-Akt: Phosphorylated protein kinase B; PD-L1: Programmed cell Death Ligand 1; PLR : Platelet-lymphocyte ratio; PRL-3: Phosphatase of Regenerating Liver 3; pSTAT3: Phosphorylated signal transducer and activator of transcription proteins 3; Sirt1: Silent information regulator 1; SOX2: Sex-determining region Y-box 2; SPARC (osteonectin or BM-40): Secreted protein acidic and rich in cysteine; STAT3: Signal transducer and activator of transcription proteins 3; TS: Thymidylate synthase; VEGF: Vascular endothelial growth factor; VEGF: Vascular endothelial growth factor; VEGF-C: Vascular endothelial growth factor-C; VEGFR-3: Vascular endothelial growth factor receptors 3; ZEB1: (TCF8, AREB6 or Zfhx1a) Zinc fnger E-box binding homeobox 1; ZEB2: (SIP1, HSPC082 and Zfhx1b) Zinc fnger E-box binding homeobox 2.
